# LAT1/SLC7A5‐mediated amino acid uptake is regulated by redox signals triggered by formyl‐peptide receptor 2

**DOI:** 10.1111/febs.70370

**Published:** 2025-12-21

**Authors:** Myrhiam Cassese, Chiara Brignola, Stefano Marrone, Gabriella Esposito, Rosario Ammendola, Fabio Cattaneo

**Affiliations:** ^1^ Department of Molecular Medicine and Medical Biotechnology University of Naples Federico II Naples Italy

**Keywords:** formyl peptide receptor 2, LAT1/SLC7A5, miR‐126, NADPH oxidase, reactive oxygen species, SLC3A2/CD98

## Abstract

The rewiring of amino acid (AA) metabolism is a key characteristic of cancer metabolism. Cells can only synthesize nonessential AAs, but if the demands of highly proliferating cells don't meet the endogenous synthesis capacity, AAs must be acquired from outside. SLC7 belongs to the solute carrier transporters (SLC) superfamily and acts as a passive facilitative or secondary AA active transporter. LAT1/SLC7A5 acts as an antiporter that mediates the influx of several AAs into cells in exchange for the efflux of intracellular substrates. In tumor cells, LAT1/SLC7A5 overexpression is closely associated with proliferation, invasion, metastasis, and poor clinical prognosis. Formyl peptide receptor 2 (FPR2) belongs to the FPR family of GPCRs. Its activation regulates several biological processes and triggers NADPH oxidase assembly and, consequently, reactive oxygen species (ROS) generation. FPR2 stimulation also induces an increase in SLC1A5/ASCT2 and SLC7A11/xCT expression, which correlates with enhanced glutamine and cystine uptake, respectively. Herein, we analyze the LAT1/SLC7A5‐mediated uptake of several essential AAs in FPR2‐stimulated CaLu‐6 and HCC1937 cells and prove: (i) the redox regulation of both LAT1/SLC7A5 and 4F2hc/SLC3A2/CD98, which form a heterodimer on the plasma membrane; (ii) the redox activation of the mTOR pathway and, in turn, of S6K1 and 4E‐BP1, which stimulate protein synthesis; (iii) c‐Myc and miR‐126 regulation, which control LAT1/SLC7A5 synthesis at the transcriptional and post‐transcriptional level, respectively. These findings provide new approaches for the development of novel therapeutic strategies for the treatment of human cancers.

## Graphical abstract

FPR2 activation mediates NOX‐dependent LAT1 expression and, in turn, promotes mTORC1 signaling. Upon stimulation with WKYMVm, the FPR2‐NOX2‐ROS axis upregulates LAT1 via increased c‐Myc phosphorylation and decreased miR‐126. Additionally, it promotes CD98 translocation to the plasma membrane. The resulting rise in leucine uptake activates mTORC1, leading to phosphorylation of S6K and 4E‐BP1.

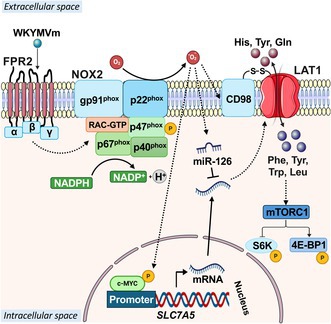

**Abbreviations**
4E‐BP1: eukaryotic translation initiation factor 4E‐binding protein 1
BCAA: branched‐chain amino acids
FPR: formyl peptide receptor
GPCR: G protein‐coupled receptor
GSH: reduced glutathione
LAT: L‐type amino acid transporter
LNAA: large neutral amino acids
mTORC: mechanistic target of rapamycin complex
NOX: NADPH oxidase
ROS: reactive oxygen species
S6K: ribosomal protein S6 kinase
SLC: solute carrier
TKR: tyrosine kinase receptor

## Introduction

Malignant cells reprogram their metabolic pathways to ensure rapid and continuous proliferation, which requires an increased demand for energy and biosynthetic precursors. Cell‐surface receptors, such as G‐protein‐coupled receptors (GPCRs) and tyrosine‐kinase receptors (TKRs), have emerged as cancer drivers that regulate many biological processes, including cell metabolism and the metabolic adaptation of cancer cells [1, 2]. Among the metabolic alterations occurring in malignant cells, amino acid (AA) metabolism rewiring represents a key hallmark of cancer metabolism. AAs are used as building blocks for protein and nucleic acid synthesis, as precursors to fuel the tricarboxylic acid cycle (TCA) for energy production, as modulators of epigenetic processes, and as activators of essential signaling pathways that promote tumor growth, invasion, and metastasis [3].

The superfamily of solute carrier transporters (SLCs) includes 65 subfamilies, totaling 458 members commonly expressed on the surface of cell membranes, endoplasmic reticulum, mitochondria, lysosomes, and peroxisomes, and involved in the transport of a broad range of substrates, such as sugars, AAs, lipids, vitamins, organic anions, and metal ions [4]. SLC1, SLC3, SLC6, SLC7, SLC36, SLC38, and SLC43 act as passive facilitative or secondary AA active transporters. Branched‐chain AAs (BCAAs), aromatic AAs, large neutral AAs (LNAAs), and definite essential AAs are transported into cells by members of the Na^+^‐independent L‐type AA transporter (LAT) family, which includes LAT1 (SLC7A5), LAT2 (SLC7A8), LAT3 (SLC43A1), and LAT4 (SLC43A2) [5]. LAT1 acts as an antiporter that mediates the influx of leucine, isoleucine, phenylalanine, methionine, histidine, tryptophan, valine, and tyrosine into cells in exchange for the efflux of intracellular substrates [6].

In tumor cells, LAT1 overexpression is closely associated with proliferation, invasion, metastasis, and poor clinical prognosis [7, 8]. LAT1/SLC7A5 expression is controlled at the transcriptional level by c‐Myc [9], HIF2α [10], and NOTCH [11], and at the post‐transcriptional level by miR‐126 [12]. LAT1/SLC7A5 and 4F2 cell‐surface antigen heavy chain (4F2hc and SLC3A2, also known as CD98) form a heterodimer via a disulfide bond on the plasma membrane [13], which is essential for the proper or stable plasma membrane localization of LAT1.

Formyl peptide receptor 2 (FPR2) is a member of the FPR family of GPCRs. Its activation elicits either inflammatory or anti‐inflammatory responses, depending on the nature of the agonist and on the different receptor domains they use [14], thus regulating several biological processes, such as cell proliferation, migration, and invasion [15]. FPR2 stimulation triggers the phosphorylation of several signaling and nonsignaling molecules [16, 17], including the cytosolic p47^phox^ subunit of NADPH oxidase (NOX) enzyme, whose phosphorylation is required for NOX assembly and, consequently, for reactive oxygen species (ROS) generation [18, 19, 20]. FPR2 is also expressed at the nuclear level, where its stimulation enhances phosphorylation and subsequent activation of specific transcription factors [21].

We demonstrated that FPR2 stimulation by its agonist WKYMVm induces a time‐dependent increase in SLC1A5/ASCT2 expression that correlates with an increased uptake of glutamine [22]. Cancer cells use glutamine as a source of carbon and nitrogen atoms to support the biosynthesis of macromolecules, energy processes, and cell homeostasis. We also proved that FPR2 modulates SLC7A11/xCT expression and, in turn, the cellular uptake of cystine in exchange for intracellular glutamate, by promoting the nuclear translocation of the transcription factor Nrf2 [23]. Imported extracellular cystine is reduced to cysteine in the cytosol and participates, among others, in the biosynthesis of GSH, which is required to sustain cellular redox homeostasis [23].

In a phosphoproteomic analysis performed on the human CaLu‐6 epithelial carcinoma cell line stimulated with an FPR2 agonist, we demonstrated that redox environment modifications regulated the phosphorylation of several proteins, mostly involved in diverse aspects of cellular metabolic processes, including primary metabolism [16, 17]. Furthermore, by utilizing a metabolomic approach, we identified several metabolic pathways activated in WKYMVm‐stimulated CaLu‐6 cells, including the pentose phosphate pathway, TCA, nucleotide synthesis, and AA metabolism [22, 24].

Herein, we analyze the regulation of AAs uptake in FPR2‐stimulated CaLu‐6 and HCC1937 cells and demonstrate that LAT1/SLC7A5 antiporter expression and downstream pathways are controlled by redox signaling triggered by FPR2.

## Results

### FPR2 stimulates LAT1/SLC7A5 overexpression in CaLu‐6 and HCC1937 cells

**Fig. 1:**
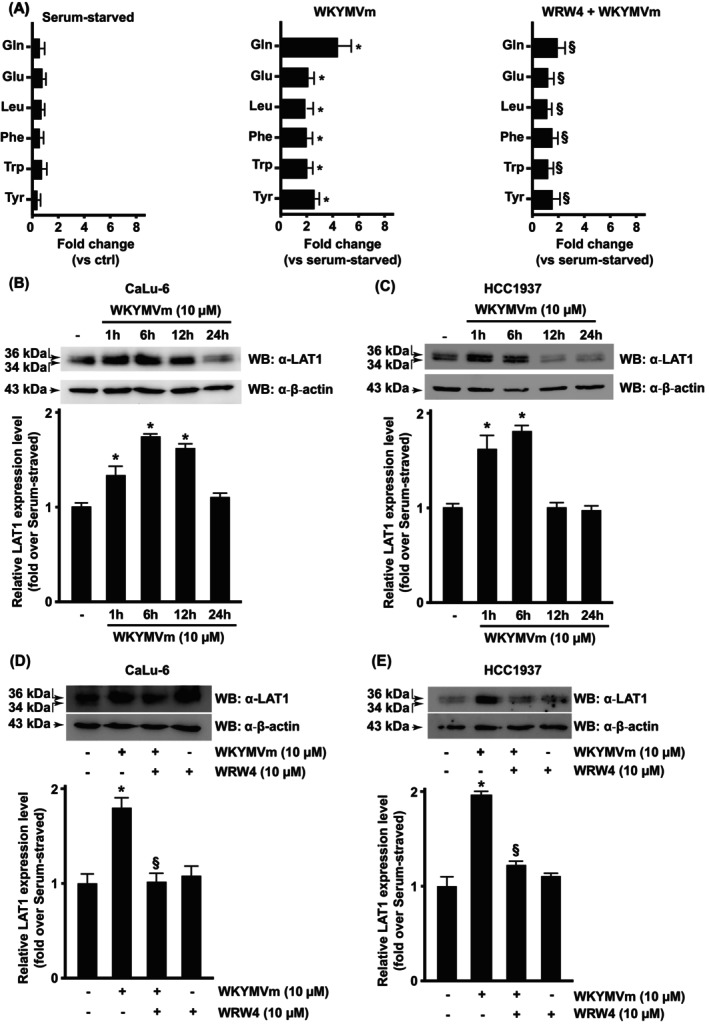
Formyl peptide receptor 2 (FPR2) stimulation promotes amino acid (AA) metabolism. (A) Metabolomic analysis of 24 h serum‐starved CaLu‐6 cells, stimulated or not with 10 μm WKYMVm, a FPR2 agonist, for 1 h in the presence or absence of 10 μm WRWWWW (WRW4), a FPR2 antagonist. (B–E) FPR2 stimulation elicits LAT1 expression. (B) CaLu‐6 cells and (C) HCC1937 cells were serum‐starved for 24 h and then stimulated for 1, 6, 12, or 24 h with WKYMVm. (D) CaLu‐6 cells and (E) HCC1937 cells were preincubated for 15 min with WRW4 before the stimulation with WKYMVm for 6 h. Fifty micrograms of whole lysates were resolved on 10% SDS/PAGE and incubated with an anti‐LAT1 antibody (α‐LAT1). An anti‐β‐actin antibody (α‐β‐actin) was used as a control for protein loading. Data are representative of three different experiments mean. Error bars indicate ± SEM. *P < 0.05 compared with unstimulated cells [one‐way analysis of variance (ANOVA)]. ^§^ *P* < 0.05 compared with stimulated cells [one‐way analysis of variance (ANOVA)].

In a metabolomic analysis performed in WKYMVm‐stimulated CaLu‐6 cells, we observed enhanced concentrations of some BCAAs, LNAAs, and aromatic AAs, namely glutamine, glutamate, leucine, phenylalanine, tryptophan, and tyrosine, and this increase was prevented by the preincubation with the peptide WRWWWW (WRW4), a selective FPR2 antagonist [22] (Fig. 1A). Previously, we proved that the glutamine transporter ASCT2 and the antiporter cystine/glutamate SLC7A11/xCT were both overexpressed in FPR2‐stimulated cells [22, 23]. Therefore, to identify pathways that control the cellular uptake of BCAAs, LNAAs, and aromatic AAs in response to WKYMVm stimulation, we analyzed the levels of LAT1/SLC7A5 protein, which were indeed upregulated in CaLu‐6 (Fig. 1B) and in HCC1937 cells (Fig. 1C) compared with the untreated cells. Preincubation with WRW4 prevents LAT1/SLC7A5 upregulation (Fig. 1D,E). Once internalized, leucine is catabolized to form acetoacetate and acetyl‐CoA, which can fuel the TCA cycle to support cellular energy metabolism or can be used as a precursor for lipogenesis. Phenylalanine and tyrosine share the same metabolic fate. Phenylalanine is hydroxylated into tyrosine, which is first transaminated into p‐hydroxyphenyl pyruvate and then converted, after several steps, into acetoacetate and fumarate, which can fuel the TCA cycle. The metabolic pathway of tryptophan shows many branch points, and the end products obtained after several reactions are glutaryl‐CoA and then acetoacetyl‐CoA.

### FPR2‐dependent LAT1/SLC7A5A expression requires a functional NOX

The increased proliferation rate of cancer cells is fueled by enhanced energy metabolism, which, in turn, results in modifications of the redox environment. Low levels of ROS can be helpful in promoting cell signaling and maintaining cellular homeostasis [25], but enhanced or imbalanced ROS production in tumor cells leads to oxidative stress, which can be a crucial factor in cancer initiation and progression [26, 27]. However, different antioxidant defense mechanisms, including non‐thiol‐dependent enzymes and thiol‐dependent antioxidant systems [28, 29], preserve the reducing cellular environment. Cancer cell metabolism is also regulated by the peculiar activation or expression of NOXs, which generate ROS in response to several stimuli [22, 24, 30, 31]. Previously, we proved that, in several cell types, FPR2 stimulation with its cognate agonists triggers p47^phox^ phosphorylation and, in turn, NOX activation through ERK‐, PKCα‐, and PKCδ‐dependent pathways [18, 19, 20].

**Fig. 2:**
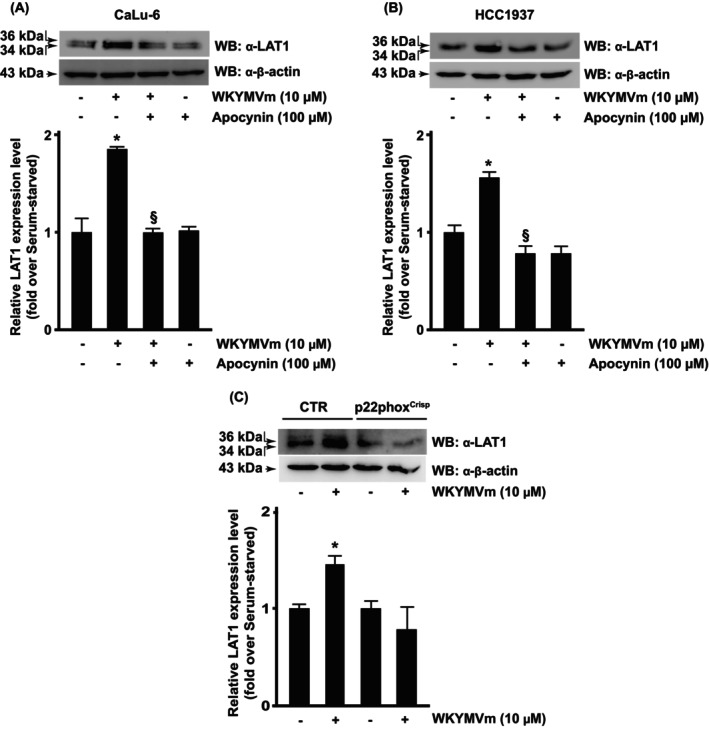
Formyl peptide receptor 2 (FPR2)‐mediated LAT1 overexpression requires NADPH oxidase (NOX) activation. Serum‐starved (A) CaLu‐6 cells and (B) HCC1937 cells were pretreated for 2 h with apocynin before 6 h stimulation of FPR2 with its agonist WKYMVm. (C) CaLu‐6‐control^Crispr/Cas9^ cells (CTR) and p22phox^Crispr/Cas9^ (p22phox^Crispr^) cells were serum‐starved for 24 h and then stimulated for 6 h with WKYMVm. Fifty micrograms of whole lysates were resolved on 10% SDS/PAGE and then incubated with an anti‐LAT1 antibody (α‐LAT1). An anti‐β‐actin antibody (α‐β‐actin) was used as a control for protein loading. Data are representative of three different experiments. Error bars indicate ± SEM. *P < 0.05 compared with unstimulated cells [one‐way analysis of variance (ANOVA)]. ^§^ *P* < 0.05 compared with stimulated cells [one‐way analysis of variance (ANOVA)].

We investigated the role of NOX‐generated ROS in FPR2‐dependent LAT1/SLC7A5 overexpression by preincubating CaLu‐6 and HCC1937 cells with apocynin before WKYMVm stimulation. Apocynin prevents the assembly of the cytosolic p47^phox^ subunit with the membrane, thus inhibiting NOX activity [32]. Obtained results showed that blocking NOX functions prevented LAT1/SLC7A5 upregulation (Fig. 2A,B). Furthermore, we modified the genome of CaLu‐6 cells by using a CRISPR/Cas9 editing tool to express a nonfunctional form of p22^phox^, the membrane partner of the NOX catalytic core gp91^phox^ (p22phox^Crispr/Cas9^) [33], and observed that WKYMVm fails to induce LAT1/SLC7A5 overexpression in these edited cells (Fig. 2C). These findings demonstrate that the FPR2‐induced LAT1/SLC7A5 overexpression depends on a functional NADPH oxidase.

### FPR2 signaling regulates 4F2hc/CD98/SLC3A2 expression in cancer cells

4F2hc, also known as CD98, is a type II transmembrane glycoprotein encoded by the solute carrier family 3‐member 2 (*SLC3A2*) gene located on chromosome 11. It is covalently linked to several light chains of AA transport systems, such as LAT1, LAT2, SLC7A11/xCT, and Ask‐type AA transporter, through disulfide bonds and electrostatic interactions [34], which are required for maintaining their stability and proper localization on the plasma membrane. The heterodimeric proteins act as chaperones for some AA transporters, facilitating their recruitment to the plasma membrane. Cancer cells can upregulate their nutritional or antioxidant capacity by overexpressing CD98/LAT1 or CD98/xCT, respectively [35, 36], and poor prognosis in malignant transformation and progression correlates with the overexpression of these two AA transporters [37, 38].

**Fig. 3:**
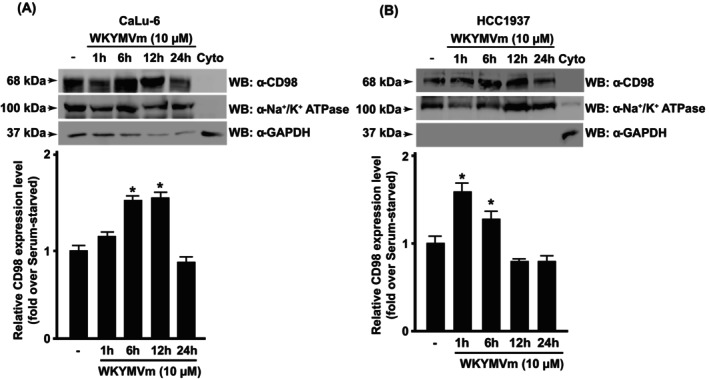
CD98 membrane translocation depends on formyl peptide receptor 2 (FPR2) activation. (A) CaLu‐6 cells and (B) HCC1937 cells were serum‐starved for 24 h and then stimulated for 1, 6, 12, or 24 h with WKYMVm, a FPR2 agonist. Fifty micrograms of membrane lysates were resolved on 10% SDS/PAGE and then incubated with an anti‐CD98 antibody (α‐CD98). An anti‐Na^+^/K^+^ ATPase antibody (α‐Na^+^/K^+^ ATPase) was used as a control for membrane protein loading. Fifty micrograms of a cytosolic fraction (Cyto) were loaded, and an anti‐GAPDH antibody (α‐GAPDH) was used as a control of cytosolic proteins. Data are representative of three different experiments. Error bars indicate ± SEM. *P < 0.05 compared with unstimulated cells [one‐way analysis of variance (ANOVA)]. ^§^ *P* < 0.05 compared with stimulated cells [one‐way analysis of variance (ANOVA)].

**Fig. 4:**
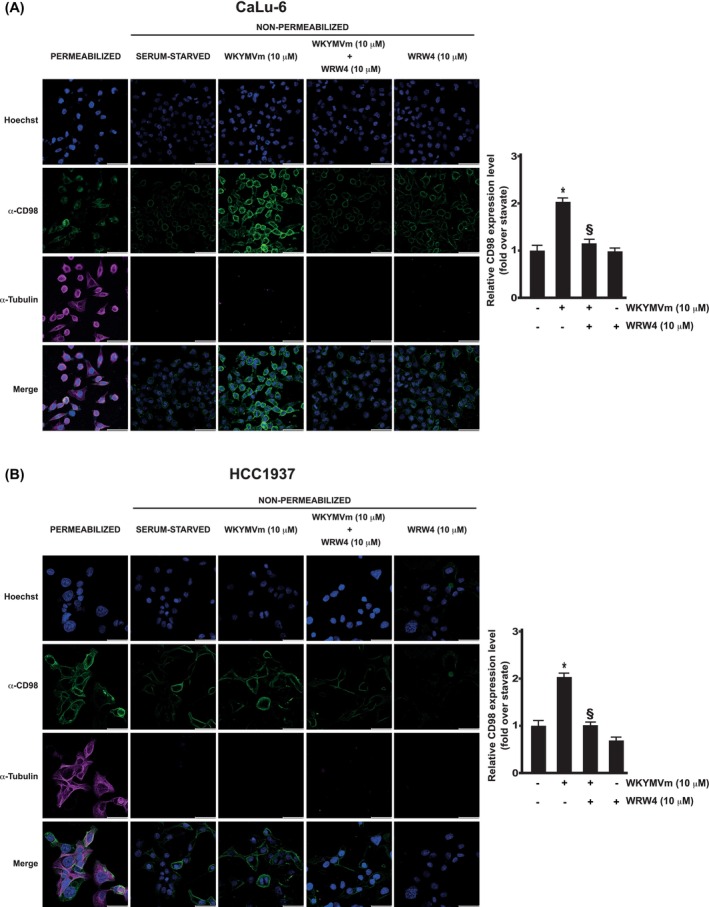
Formyl peptide receptor 2 (FPR2) stimulation with its agonist WKYMVm promotes membrane translocation of CD98. Serum‐starved CaLu‐6 (A) and HCC1937 cells (B) were stimulated with WKYMVm for 6 h or preincubated for 15 min with WRWWWW (WRW4), a FPR2 antagonist, before stimulation. Cells were incubated with an anti‐CD98 primary antibody, followed by incubation with an anti‐rabbit Alexa Fluor 488‐conjugated secondary antibody (CD98, green signal). To visualize vital nuclei, cells were also incubated with Hoechst 33342 (Hoechst, blue signal). As an internal control, cytoplasmic α‐tubulin was simultaneously detected with an anti‐α‐tubulin primary antibody, followed by incubation with an anti‐mouse Alexa Fluor 594‐conjugated secondary antibody (α‐tubulin, red signal). Images were captured and merged using the THUNDER Imager 3D Cell Culture system (merge). Scale bar: 50 μm. Data are representative of five different experiments. Error bars indicate ± SEM. *P < 0.05 compared with unstimulated cells [one‐way analysis of variance (ANOVA)]. ^§^ *P* < 0.05 compared with stimulated cells [one‐way analysis of variance (ANOVA)].

In time‐course experiments, we observed that FPR2 stimulation induces a time‐regulated increase of CD98 expression on cellular membranes, both in CaLu‐6 (Fig. 3A) and in HCC1937 cells (Fig. 3B). These data were confirmed by immunofluorescence experiments, in which an enhanced expression of CD98 was observed upon 6 h of FPR2 stimulation, both in CaLu‐6 (Fig. 4A) and HCC1937 cells (Fig. 4B). These results demonstrate that FPR2‐related signaling pathways influence the expression level of both components of the CD98/LAT1 heterodimer.

### FPR2‐dependent leucine uptake activates mTOR pathway

Tumor growth and proliferation are affected by the LAT1/SLC7A5‐mediated uptake of leucine and other AAs, which triggers the activation of multiple signals, including the mammalian targets of rapamycin (mTOR) pathway [5]. mTOR belongs to the phosphoinositide‐3‐kinase‐related kinase (PIKK) family, which shows both serine–threonine kinase and tyrosine kinase activity. The two major complexes activated by the mTOR signaling cascade are mTOR complex 1 and 2 (mTORC1 and mTORC2). In mTORC1, mTOR is complexed with regulatory associated protein of mTOR (RAPTOR), mammalian lethal with SEC13 protein 8 (mLST8), 40 kDa proline‐rich Akt substrate (PRAS40), and DEP‐domain‐containing mTOR‐interacting protein (DEPTOR) [39]. In response to several stimuli, mTORC1 catalyzes the phosphorylation of molecules that play a critical role in the stimulation of protein synthesis, such as S6 kinase 1 (S6K1) and eukaryotic translation initiation factor 4E‐binding protein 1 (4E‐BP1) [40], while mTORC2 phosphorylates AKT to promote cell survival [41, 42]. The mechanisms by which leucine is sensed and activates mTORC1 remain unclear. A leucyl‐tRNA synthetase (LRS) can detect the levels of leucine in the cell and then catalyze the ATP‐dependent ligation of L‐leucine to leucyl‐tRNA during protein synthesis [43, 44]. LRS, in turn, interacts with and activates the Rag GTPase complexes, which are Ras‐related small GTP‐binding proteins. Rag GTPase activation is essential only for leucine‐ or arginine‐activated mTORC1 signaling [45]. LRS can also bind RAPTOR and activate mTORC1 signaling [44, 45]. S6K1 can be phosphorylated by mTORC1 at multiple Thr and Ser residues in a hierarchical sequence, activating, in turn, the S6 protein on the 40S ribosomal protein, which is involved in protein synthesis, proliferation, and cell survival.

**Fig. 5:**
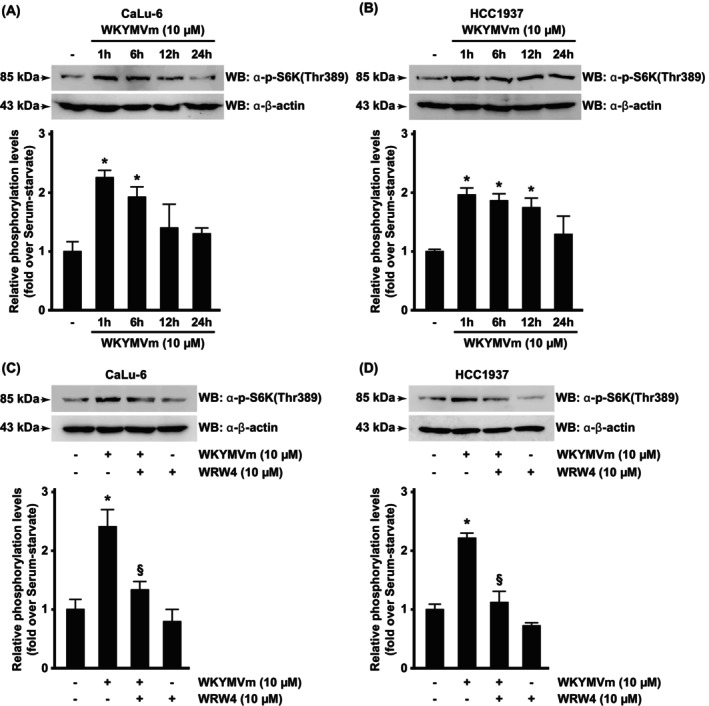
Formyl peptide receptor 2 (FPR2) signaling elicits S6 Kinase phosphorylation. Serum‐starved (A) CaLu‐6 and (B) HCC1937 cells were stimulated for the indicated times with the FPR2 agonist WKYMVm or preincubated with the FPR2 antagonist WRWWWW (WRW4) before WKYMVm stimulation (C, D). Fifty micrograms of whole lysates were resolved on 10% SDS/PAGE and incubated with an anti‐phospho‐p70 S6 Kinase (Thr389) antibody [α‐p‐S6K(Thr389)]. An anti‐β‐actin antibody (α‐β‐actin) was used as a control of protein loading. Data are representative of four different experiments. Error bars indicate ± SEM. *P < 0.05 compared with unstimulated cells [one‐way analysis of variance (ANOVA)]. ^§^ *P* < 0.05 compared with stimulated cells [one‐way analysis of variance (ANOVA)].

We investigated the FPR2‐dependent S6K1 phosphorylation by using a phospho‐antibody targeting the critical phosphorylated Thr^389^ residue, and we observed a time‐dependent increase of S6K1^Thr389^ phosphorylation in both CaLu‐6 (Fig. 5A) and HCC1937 (Fig. 5B) cell lines. Preincubation with the FPR2 antagonist completely prevents this phosphorylation (Fig. 5C,D).

**Fig. 6:**
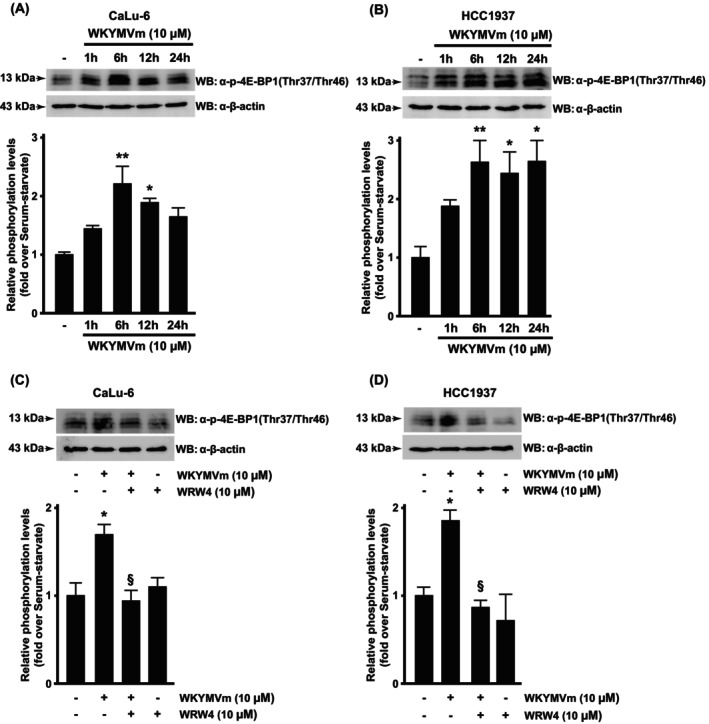
Formyl peptide receptor 2 (FPR2) stimulation triggers 4E‐BP1 phosphorylation. Serum‐starved (A) CaLu‐6 and (B) HCC1937 cells were stimulated with WKYMVm, a FPR2 agonist, for the indicated times. Cells were also preincubated with WRWWWW (WRW4), a FPR2 antagonist, before WKYMVm stimulation (C and D). Fifty micrograms of whole lysates were resolved on 15% SDS/PAGE and incubated with an anti‐phospho‐4E‐BP1(Thr37/Thr46) antibody [α‐p‐4E‐BP1(Thr37/Thr46)]. An anti‐β‐actin antibody (α‐β‐actin) was used as a control of protein loading. Data are representative of four different experiments. Error bars indicate ± SEM. *P < 0.05, **P < 0.01 compared with unstimulated cells [one‐way analysis of variance (ANOVA)]. ^§^ *P* < 0.05 compared with stimulated cells [one‐way analysis of variance (ANOVA)].

The eukaryotic translation initiation factor (eIF) 4E recruits 40S ribosomal subunits to the 5′ end of mRNA. Assembly of the eIF4E complex is prevented by the binding with the eIF4E‐binding protein 1 (4E‐BP1), which is regulated by phosphorylation. Hypophosphorylation of 4E‐BP1 strongly influences its binding with eIF4E, while it is significantly reduced by hyperphosphorylation. A high level of mTORC1 activity catalyzes the phosphorylation of 4E‐BP1 at Thr^37^ and Thr^46^ residues, thus increasing translation rates. We observed that WKYMVm stimulation induced 4E‐BP1^Thr37Thr46^ phosphorylation both in Calu‐6 (Fig. 6A) and HCC1937 (Fig. 6B) cells. Such phosphorylation was prevented by preincubation with WRW4 (Fig. 6C,D). Overall, these results demonstrate that the FPR2‐dependent and redox‐regulated LAT1/SLC7A5 overexpression induces an increase in leucine uptake that, in turn, activates mTORC1 and the downstream proteins S6K1 and 4E‐BP1.

### mTORC1 activation depends on NOX activity

ROS can act as second messengers in TKR transactivation and, in turn, in the activation of TKR‐dependent intracellular signaling cascades [24, 46, 47, 48]. Phosphorylation/dephosphorylation mechanisms, catalyzed by protein kinases and phosphatases, respectively, represent the most common covalent modification of proteins. Protein tyrosine phosphatases possess reactive cysteines in their catalytic domain that ROS can reversibly oxidize, thus inhibiting their dephosphorylation activity [49] and facilitating the initiation of the signaling response to extracellular stimuli mediated by TKRs [50, 51]. Signals triggered by FPR2 induce the phosphorylation of several signaling and nonsignaling proteins, thus modulating many critical intracellular functions [52]. Among these, p47^phox^ phosphorylation and the regulation of NOX activity represent an important strategy for the deliberate generation of ROS.

**Fig. 7:**
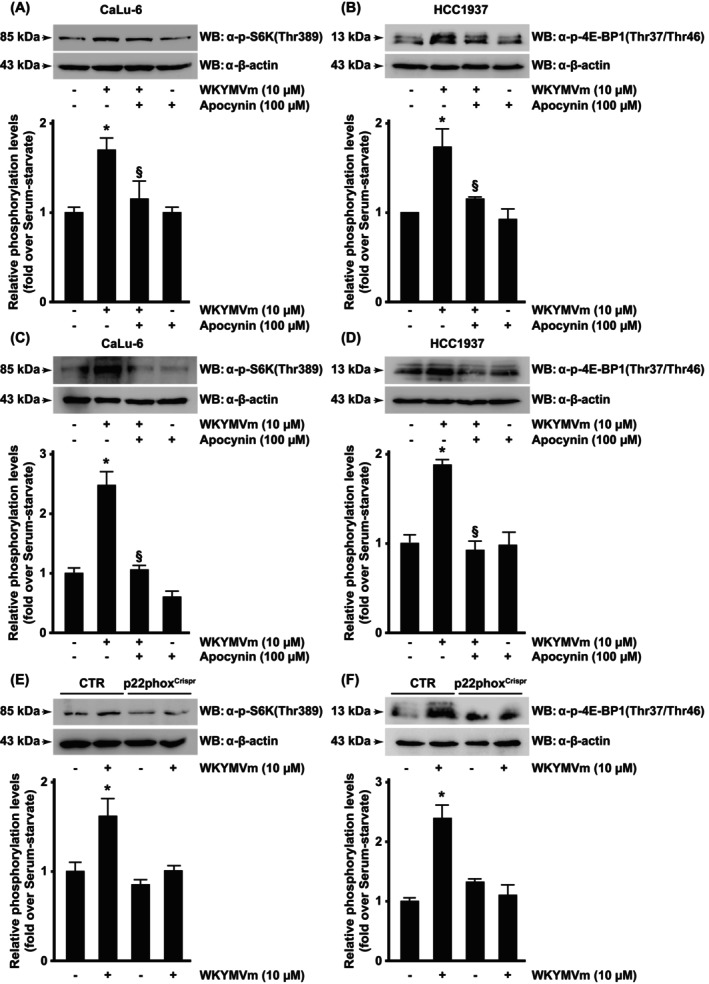
Formyl peptide receptor 2 (FPR2)‐induced phosphorylation of S6 Kinase and 4E‐BP1 depends on NADPH oxidase (NOX) activity. Serum‐starved (A and B) CaLu‐6 and (C and D) HCC1937 cells were preincubated with apocynin before FPR2 stimulation with its agonist WKYMVm. (E and F) CaLu‐6‐control^Crispr/Cas9^ cells (CTR) and p22phox^Crispr/Cas9^ (p22phox^Crispr^) cells were serum‐starved for 24 h and then stimulated for 6 h with WKYMVm. Fifty micrograms of whole lysates were resolved on 10% or 15% SDS/PAGE and incubated with an anti‐phospho‐p70 S6 Kinase(Thr389) antibody [α‐p‐S6K(Thr389)] (A, C, E), or with an anti‐phospho‐4E‐BP1(Thr37/Thr46) antibody [α‐p‐4E‐BP1(Thr37/Thr46)] (B, D, F). An anti‐β‐actin antibody (α‐β‐actin) was used as a control of protein loading. Data are representative of three different experiments. Error bars indicate ± SEM. *P < 0.05 compared with unstimulated cells [one‐way analysis of variance (ANOVA)]. ^§^ *P* < 0.05 compared with stimulated cells [one‐way analysis of variance (ANOVA)].

The RAPTOR‐mTOR complex is regulated by redox‐sensitive mechanisms. It is activated by thiol oxidants, and hence by cysteine oxidation [53], which destabilizes this interaction, whereas reducing reagents stabilize the RAPTOR‐mTOR complex, thus inhibiting the downstream pathway [54]. Therefore, to further investigate the role of the FPR2‐NOX‐ROS axis on mTORC activation, we exposed both CaLu‐6 and HCC1937 cells to WKYMVm for 6 h, preincubated or not with apocynin (Fig. 7A–D), as well as p22phox^Crispr/Cas9^ cells (Fig. 7E,F). In these three types of cells, we analyzed FPR2‐dependent S6K1^Thr389^ and 4E‐BP1^Thr37Thr46^ phosphorylation and found that they were prevented by blocking NOX function (Fig. 7A–F). Taken together, these results further indicate that NOX‐dependent ROS generation, activated by FPR2 signaling, regulates mTORC1 activity.

### FPR2 signaling regulates c‐Myc and miR‐126 expression

**Fig. 8:**
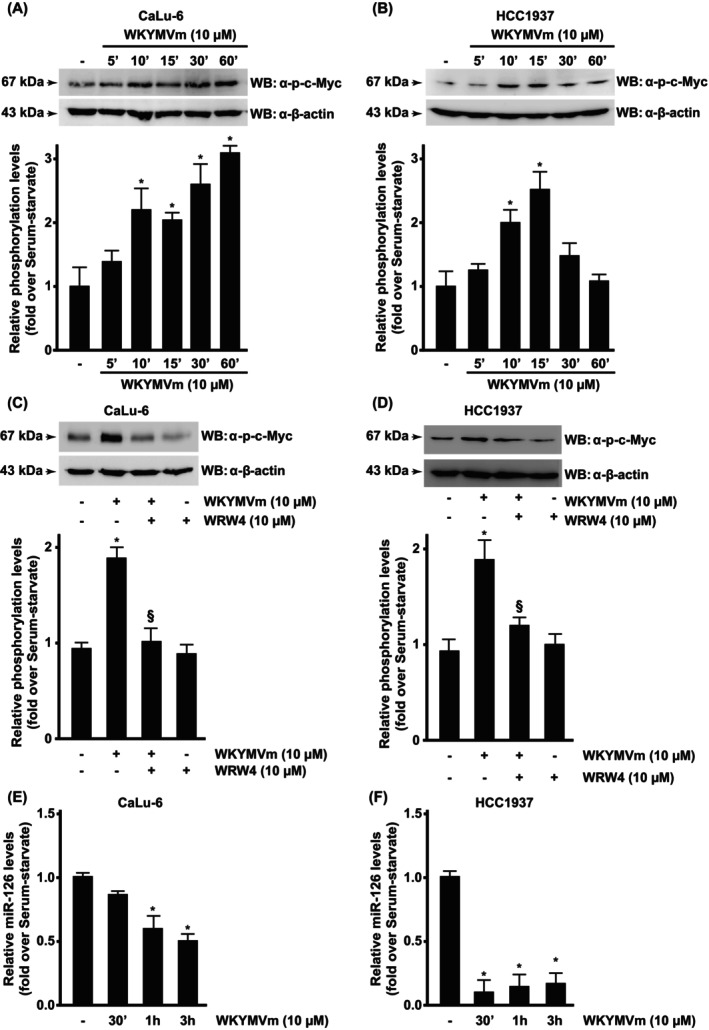
Formyl peptide receptor 2 (FPR2) stimulation regulates phospho‐c‐Myc and miR‐126 expression. (A–D) FPR2 signaling triggers c‐Myc phosphorylation. Serum‐starved (A) CaLu‐6 cells and (B) HCC1937 cells were stimulated with WKYMVm, a FPR2 agonist, for the indicated times. CaLu‐6 and HCC1937 cells were also preincubated with WRWWWW (WRW4), a FPR2 antagonist, before WKYMVm stimulation (C and D). Fifty micrograms of whole lysates were resolved on 10% SDS/PAGE and incubated with an anti‐phospho‐c‐Myc(Thr58/Ser62) antibody (α‐p‐c‐Myc). An anti‐β‐actin antibody (α‐β‐actin) was used as a control for protein loading. (E and F) FPR2 signaling regulates miR‐126 expression. (E) CaLu‐6 and (F) HCC1937 cells were stimulated with WKYMVm for the indicated times. Relative miR‐126 levels were evaluated by RT‐qPCR from total RNA. RNU6B was used as an internal control. Data are representative of four independent experiments. Error bars indicate ± SEM. *P < 0.05 compared with unstimulated cells [one‐way analysis of variance (ANOVA)]. ^§^ *P* < 0.05 compared with stimulated cells [one‐way analysis of variance (ANOVA)].

*The Myc family of* oncogenes contains three members, *c‐Myc, L‐Myc*, and *N‐Myc*, which regulate several aspects of tumor metabolism. Genes involved in rapidly synthesizing building blocks, such as ribosome biogenesis, lipid and nucleotide biosynthesis, glucose, and glutamine metabolism, are activated by Myc protein [9]. Tumor cells depend on essential AAs as the basic building blocks for macromolecular biosynthesis, and since they cannot be synthesized *de novo*, highly effective AA transporters must maximize their uptake. In cancer cells, Myc activates SLC7A5 expression, promoting effective AA uptake, which, in turn, stimulates Myc synthesis, creating a positive and connected autoregulatory circuit that strengthens Myc‐regulated oncogenic programs [9]. We observed a time‐regulated increase of c‐Myc expression in FPR2‐stimulated CaLu‐6 (Fig. 8A) and in HCC1937 (Fig. 8B), which is prevented by WRW4 preincubation (Fig. 8C,D).

MicroRNAs (miRNAs) play a crucial role in the regulation of cell proliferation in normal and tumor cells. miR‐126 has an antiproliferative effect in non‐small cell lung cancer (NSCLC) cells and other tumor types by targeting different members of the PI3K/Akt pathway [55] and SLC7A5 [12]. miR‐126 overexpression negatively affects NSCLC cell proliferation and delays the entry of these cells into the G1 phase. On the contrary, decreased miR‐126 expression contributes to high SLC7A5 expression, allowing more efficient transport of essential AAs in the rapidly proliferating NSCLC tumor cells [12]. Consistently, we observed by real‐time qPCR a decreased expression of miR‐126 in FPR2‐stimulated cells (Fig. 8E,F), which correlated with the increased LAT1/SLC7A5 expression. Taken together, these data indicate that FPR2‐mediated LAT1/SLC7A5 increase is regulated at the transcriptional level by c‐Myc and at the post‐transcriptional level by miR‐126.

## Discussion

Tumor proliferation and homeostasis are greatly influenced by AAs, which act as intermediaries between glucose, lipid, and nucleotide metabolism. Essential AAs must be obtained from external sources since they cannot be synthesized by cells that can instead synthesize nonessential AAs. Nevertheless, if the increased demands of highly proliferating cells do not meet the endogenous synthesis capacity, they must obtain AAs from outside. The plasma membrane is inaccessible to AAs because they are all hydrophilic, but several AA transporters are differentially expressed in mammalian cells in a tissue‐ and development‐specific manner. Tumor cells upregulate selective AA transporters, and blocking their function can be exploited for targeted cancer therapy.

LAT1/SLC7A5, one of the System L (Leucine‐preferring) AA transporters, is an obligatory exchanger and is highly expressed in most cancers. We observed that FPR2 stimulation induced LAT1/SLC7A5 overexpression in two tumor cell lines, thus enabling these cells to increase the uptake of leucine and other AAs (Fig. 1B–E). Notably, other members of the GPCR family, such as taste receptors (T1Rs) and GPRC6A, can sense extracellular AAs and transmit signals [56]. The heterodimer T1R1/T1R3 acts as an AA sensor responding to Leu, Ile, Phe, Met, His, Val, and other standard AAs [57]. GPRC6A can be activated by Gly, Arg, Lys, Ala, and Ser [58], triggering ERKs and PI3K‐Akt pathways and, in turn, mTORC1 activation through ERK‐dependent RAPTOR phosphorylation [59] and Akt‐dependent enhancement of Rheb‐GTP activity [60].

Redox conditions affect LAT1/SLC7A5 expression. A prolonged oxidative stress, such as hyperoxia, impairs the LAT1 function *in vitro* and its expression *in vitro* and *in vivo* [61]. We showed that short‐term and low levels of NOX‐dependent ROS generation promoted LAT1/SLC7A5 upregulation, which was prevented by the blockade of NOX functions (Fig. 2A–C). This observation is coherent with the LAT1 structure, consisting of 12 transmembrane regions inside which 12 conserved cysteine residues are present [62]. Among them, Cys^88^ and Cys^439^ residues are involved in the transporter activity, and the Cys439Ser mutant prevents either LAT1 folding and/or LAT1 insertion in the plasma membrane [63]. Furthermore, a disulfide bond between LAT1‐Cys^210^, located in an extracellular loop between transmembrane regions 3 and 4, and 4F2hc‐Cys^164^ plays a crucial role in the formation of LAT1/4F2hc heterodimer on the cellular membrane [64]. Thus, the mild oxidant environment generated by FPR2‐dependent NOX activity could promote the formation of disulfide bonds between Cys residues, hence stabilizing LAT1 structure and function on the plasmalemma.

Similarly, the RAPTOR‐mTOR pathway is regulated by redox‐sensitive mechanisms. It can occur at the cysteine residues level of the small GTPases Rheb, which can sense modifications of the cellular redox status [65]. Rheb activity is tightly regulated by Tuberous sclerosis complex 2 (TSC2) [65, 66, 67]. The serine/threonine protein kinase G‐1α (PKG1α) phosphorylates and activates TSC2 to suppress mTORC1 activity. Oxidation of PKG1α at the Cys^42^ residue reduces its ability to phosphorylate TSC2 at the Ser^1365^ residue, resulting in increased mTORC1 activity [68].

Activation of mTORC1 promotes anabolic processes, including mRNA translation, ribosome biogenesis, and protein synthesis, and inhibits catabolic processes [69]. Different signaling inputs are involved in the mTORC1 regulation. TKR activation contributes to oncogenic stimulation of the PI3K/Akt/mTOR pathway [70], triggering phosphoinositide 3‐kinase (PI3K) activation and, in turn, phosphatidylinositol‐3,4,5‐trisphosphate (PIP3) generation. This activates the protein kinase B (Akt), which inhibits TSC2 by phosphorylation, thus removing its inhibitory effect on mTORC1. GPCR stimulation also triggers the PI3K/Akt pathway. We previously demonstrated that FPR2 activation stimulated TKRs transactivation and that NOX‐dependent ROS generation played a role as a second messenger in this molecular mechanism and in the modulation of TKR‐dependent intracellular signaling cascades [46, 48].

ROS may have a positive or negative impact on mTOR, depending on their concentration. Hydrogen peroxide (H_2_O_2_) generates potent oxidative stress and can activate or inhibit mTORC1 or mTORC1 downstream targets (S6K1 or 4E‐BP1), depending on exposure length, dose/concentration, and cell type. In HEK293 cells, H_2_O_2_ induces AMPK phosphorylation, which, in turn, phosphorylates RAPTOR and mTORC1 [71]. In contrast, in neurons, mTOR signaling is prevented by H_2_O_2_‐mediated AMPK activation, while it is stimulated by antioxidants [72]. In HEK293 cells, the oxidizing agent diamide triggers the dissociation between RAPTOR and mTOR and, in turn, S6K1 phosphorylation, which can be restored by thioredoxin [73]. Consistently, we observed that low concentrations of NOX‐dependent ROS supported mTORC1 activation and, in turn, S6K1 and 4E‐BP1 phosphorylation, which was prevented by the blockade of NOX functions (Figs 5A–D, 6A–D).

The Myc oncoproteins are master regulators of metabolic reprogramming by controlling distinct sets of genes through binding to specific E‐box cis‐elements [74]. Myc selectively activates *Slc7a5* transcription, enabling effective AA import, and LAT1/SLC7A5 depletion inhibits Myc expression, metabolic reprogramming, and tumor cell growth [9]. This Myc‐SLC7A5 signaling circuit drives AA metabolism, Myc deregulation, and tumorigenesis. Accordingly, in time‐course experiments, we showed correlated overexpression of c‐Myc and SLC7A5 in FPR2‐stimulated cells (Figs 1B,C, 7A,B). At the post‐transcriptional level, SLC7A5 is regulated by miR‐126, which shows multiple functions, depending on the cell type and the cellular environment. Glutamine–leucine exchange by SLC7A5 activates mTOR, which, in turn, phosphorylates S6K1 and 4E‐BP1, resulting in the production of growth‐promoting proteins. The positive feedback between SLC7A5 and mTOR is enhanced when miR‐126 is silenced [12], thus contributing to the proliferative potential of tumor cells. Consistently, we showed that miR‐126 was downregulated in FPR2‐stimulated cancer cells (Fig. 8E,F).

Regulatory mechanisms of tumor AA metabolism are involved in growth, epigenetic modification, immunity, and ferroptosis, and many strategies for cancer therapy focused on these processes have been developed. Among them, targeting enzymes involved in cancer AA metabolism via small molecule inhibitors or RNAi‐targeting approaches, pharmacologically inhibiting AA transporters, and specific AA deprivation by drugs have aroused great interest.

The development of novel drugs for cancer therapy is greatly facilitated by the identification of highly successful and specific LAT1/SLC7A5 regulators. Thus, the observation that FPR2 stimulation and NOX‐dependent ROS generation modulate LAT1/SLC7A5 expression identifies alternative pathways controlling leucine and other essential AAs uptake and, hence, novel approaches to develop strategies for the treatment of human cancers.

## Materials and methods

### Cell cultures and reagents

Anaplastic lung cancer CaLu‐6 (ATCC HTB‐56™, RRID: CVCL_0236) and ductal carcinoma HCC1937 (ATCC CRL‐2336™, RRID: CVCL_0290) cell lines were from ATCC (American Type Cell Collection, Rockville, MD, USA). CaLu‐6 cells were cultured in Dulbecco's Modified Eagle Medium (DMEM) supplemented with 10% fetal bovine serum (FBS), while HCC1937 cells were grown in Roswell Park Memorial Institute (RPMI) medium, also supplemented with 10% FBS. Both cell lines were maintained in an incubator with a humidified atmosphere with 5% CO_2_ at 37 °C. When cells reached 70% confluence, they were serum‐starved for 24 h and subsequently stimulated, or not, with 10 μm WKYMVm (Primm, Milan, Italy) for various times. In additional experiments, cells were pretreated with 10 μm WRW4 (Primm, Milan, Italy) for 15 min or 100 μm apocynin (Sigma Chemical, St. Louis, MO, USA) for 2 h prior to WKYMVm incubation. All experiments were performed in mycoplasma‐free cells.

### p22phox^Crispr^ ^/Cas9^ Double‐Nickase CaLu‐6 cells

The p22phox^Crispr/Cas9^ cells were generated as described previously [33]. Briefly, CaLu‐6 cells were transfected with a Double Nickase Plasmid (Santa Cruz Biotechnology, Irvine, CA, USA), according to the manufacturer's instructions. Transfected cells were selected using puromycin, and p22phox expression was assessed by western blotting. The validated p22phox knockout clones were collected to generate the p22phox^CRISPR/Cas9^ cell line.

### Protein extraction and western blot

Proteins were purified from 24 h serum‐starved CaLu‐6, p22phox^Crispr/Cas9^ CaLu‐6, and HCC1937 cells stimulated or not with 10 μm WKYMVm, in the presence or absence of selective inhibitors, as described above. Whole lysates were obtained by scraping the cells with ice‐cold RIPA buffer containing 50 mm Tris–HCl, pH 7.4, 150 mm NaCl, 1% NP‐40, 1 mm EDTA, 0.25% sodium deoxycholate, 1 mm NaF, 10 μm Na_3_VO_4_, 1 mm phenylmethylsulfonylfluoride, and a protease inhibitor cocktail (10 μg·mL^−1^ aprotinin, 10 μg·mL^−1^ pepstatin, and 10 μg·mL^−1^ leupeptin). Cell lysates were incubated under constant agitation at 4 °C for 45 min and centrifuged at 14 000 rpm for 15 min at 4 °C.

For membrane lysates, cells were lysed in ice‐cold hypotonic buffer (Buffer I) containing 20 mm MES pH 6.0, 2 mm MgCl_2_, 5 mm KCl, 1 mm phenyl‐methyl‐sulfonyl‐fluoride, and the protease inhibitor cocktail. The lysates were centrifuged at 2100 rpm for 10 min at 4 °C, separating the cytosolic fraction in the supernatant and the membrane fraction in the pellet. The pellet was washed twice in phosphate‐buffered saline (PBS) and incubated overnight at 4 °C under constant agitation in a buffer (Buffer II) containing 20 mm MES pH 6.0, 2 mm MgCl_2_, 5 mm KCl, 1 mm phenyl‐methyl‐sulfonyl‐fluoride, 1% Triton X100, and the protease inhibitor cocktail. Protein concentration was determined using the Bradford protein assay (Bio‐Rad, Hercules, CA, USA). Primary antibodies were used at a 1:1000 dilution, including anti‐LAT1/SLC7A5, anti‐CD98, anti‐phospho‐4E‐BP1 (Thr37/Thr46) from GeneTex (Alton Pkwy, Irvine, CA, USA), and anti‐phospho‐p70 S6 Kinase (Thr389) from Sigma‐Aldrich (Winston Park Drive, Oakville, ON, Canada). Anti‐Na^+^/K^+^ ATPase, anti‐GAPDH, and anti‐β‐actin were from Santa Cruz Biotechnology (Alton Pkwy, Irvine, CA, USA). HRP‐conjugated secondary antibodies, goat anti‐mouse and goat anti‐rabbit, were obtained from Bioss Antibodies (Woburn, MA, USA). Signals were visualized using enhanced chemiluminescence reagent (Amersham Biosciences, Little Chalfont, Buckinghamshire, UK) and quantified by densitometric analysis using a Chemidoc imaging system (Bio‐Rad, Hercules, CA, USA). Full blots are available in the Data S1. Each experiment and corresponding densitometric quantification were independently repeated at least three times.

### Immunofluorescence

Serum‐starved CaLu‐6 and HCC1937 cells, either stimulated with 10 μm WKYMVm or left untreated, in the presence or absence of WRW4, were washed with PBS containing Ca^2+^ and Mg^2+^, fixed with 4% paraformaldehyde (PFA) for 20 min at room temperature (RT), quenched with 50 mm NH₄Cl for 10 min at RT, and subsequently blocked with 1% bovine serum albumin (BSA) in PBS for 10 min. To detect cell‐surface expression of CD98, nonpermeabilized cells were incubated with an anti‐CD98 primary antibody (1:100; GeneTex, Alton Pkwy, Irvine, CA, USA) and anti‐α‐Tubulin primary antibody (1:1000; Cell Signaling, Danvers, MA, USA) as a negative control. As a control for α‐Tubulin detection, cells were permeabilized with 0.1% Triton X‐100 (Sigma, Saint Louis, MO, USA) in 1% BSA/PBS for 10 min at RT. Permeabilized cells were also incubated with anti‐CD98 and anti‐α‐Tubulin primary antibodies. Subsequently, both nonpermeabilized and permeabilized cells were washed with 1% BSA in PBS and stained with either goat anti‐rabbit Alexa Fluor 488‐conjugated (1:200; Invitrogen, Carlsbad, CA, USA) or goat anti‐mouse Alexa Fluor 594‐conjugated (1:200; Invitrogen) secondary antibodies for 45 min at RT. Nuclei were stained with Hoechst 33258 (1:3000; DOJINDO Laboratories Co., Ltd, Kumamoto, Japan) for 10 min. Finally, cells were washed with 1% BSA in PBS and imaged using a THUNDER Imager 3D Cell Culture system.

### TaqMan real‐time PCR assays

Total RNA was extracted from serum‐starved CaLu‐6 and HCC1937 cells, stimulated or not with WKYMVm in the presence or absence of WRW4, using the mirVana™ miRNA Isolation Kit with phenol (Invitrogen), according to the manufacturer's instructions. miRNA targets were reverse transcribed from total RNA using the TaqMan™ MicroRNA Reverse Transcription Kit (Applied Biosystems, Foster City, CA, USA) with a pool of RT primers from the TaqMan™ Small RNA Assays (Applied Biosystems). U6 small nuclear RNA (RNU6B) was used as the internal control. Specific primers for miR‐126 and RNU6B were obtained from the TaqMan™ MicroRNA Assays (Applied Biosystems). RT‐qPCR analysis was performed following the manufacturer's protocol. Relative expression levels were calculated using the 2^−ΔΔCt^ method.

### Statistical analysis

Differences between groups were assessed for statistical significance using the one‐way analysis of variance (ANOVA) and then determined with the least significant difference test. All data reported are representative of at least three independent experiments and are expressed as mean ± standard error mean (SEM). A *P* value of <0.05 was considered statistically significant.

## Author contributions

MC contributed to the investigation, conceptualization, methodology, data curation, validation, and data analysis. CB contributed to the investigation, conceptualization, methodology, data curation, and data analysis. SM contributed to the investigation, methodology, and data analysis. GE contributed to the conceptualization, review, editing, and validation. RA and FC contributed to the conceptualization, supervision, review, editing, and funding acquisition. MC and CB contributed equally to the work. All authors have read and approved the final version of the manuscript.

## Conflict of interest

The authors declare no conflict of interest.

## Supporting information


**Figure S1.** Full blot Figure 1B‐E. WKYMVm is a FPR2 agonist; WRWWWW (WRW4) is a FPR2 antagonist.
**Figure S2**. Full blot Figure 2A‐C. WKYMVm is a FPR2 agonist; Apocynin is selective inhibitor of p22phox. CaLu‐6‐control ^Crispr/Cas9^ cells (CTR) and p22phox ^Crispr/Cas9^ (p22phox^Crispr^).
**Figure S3**. Full blot Figure 3A and B. WKYMVm is a FPR2 agonist. Cytosolic fraction (Cyto).
**Figure S4**. Full blot Figure 5A‐D. WKYMVm is a FPR2 agonist; WRWWWW (WRW4) is a FPR2 antagonist.
**Figure S5**. Full blot Figure 6A‐D. WKYMVm is a FPR2 agonist; WRWWWW (WRW4) is a FPR2 antagonist.
**Figure S6**. Full blot Figure 7A‐D. Full blot Figure 2A‐C. WKYMVm is a FPR2 agonist; Apocynin is selective inhibitor of p22phox.
**Figure S7**. Full blot Figure 7E and F. Full blot Figure 2A‐C. WKYMVm is a FPR2 agonist; CaLu‐6‐control ^Crispr/Cas9^ cells (CTR) and p22phox ^Crispr/Cas9^ (p22phox^Crispr^).

## Data Availability

All the data presented in this study are available within the article and/or the supporting information.
