# Supplementary material for: LAT1/SLC7A5‐mediated amino acid uptake is regulated by redox signals triggered by formyl‐peptide receptor 2
Source: FEBS J. 2025 Dec 21;293(9):2637–55. doi: 10.1111/febs.70370 (PMC13147325; doi:10.1111/febs.70370)

Supplementary Figure S1

1B. WB: α-LAT1

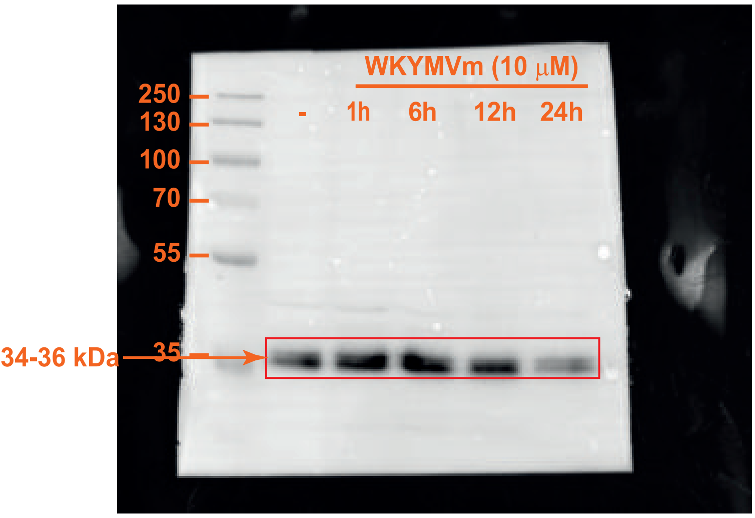

1C. WB: α-LAT1

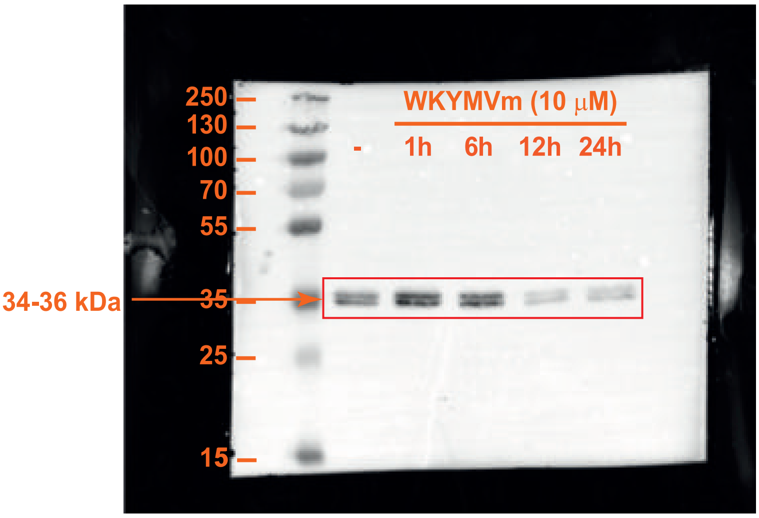

1B. WB: α-β-actin

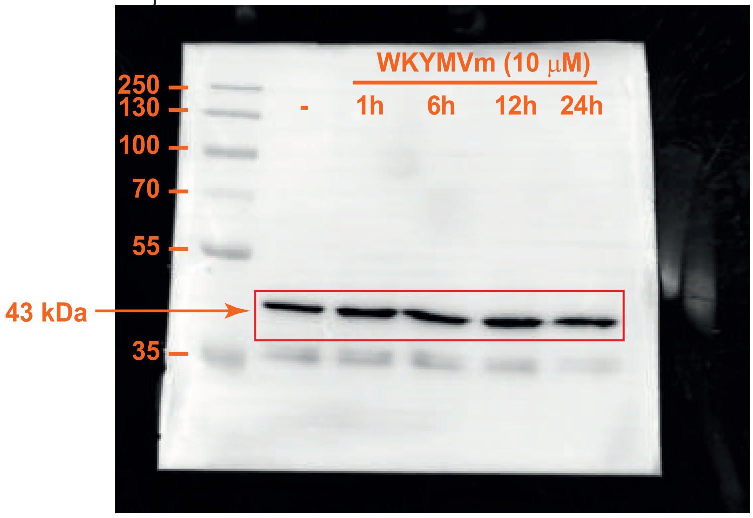

1C. WB: α-β-actin

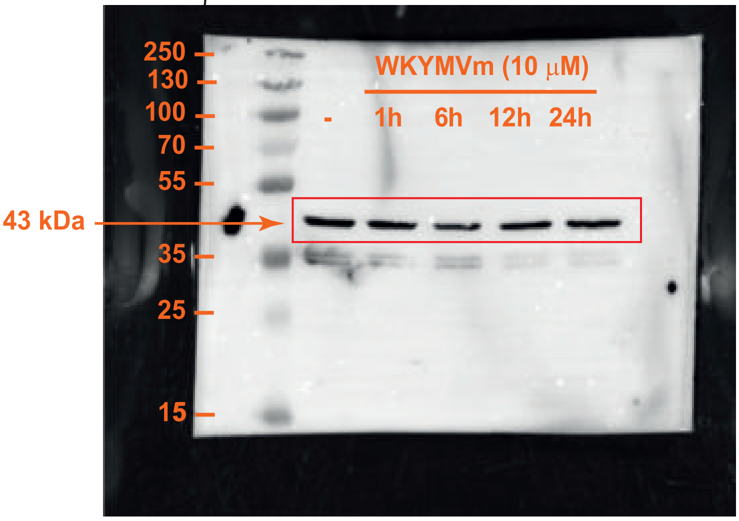

1D. WB: α-LAT1

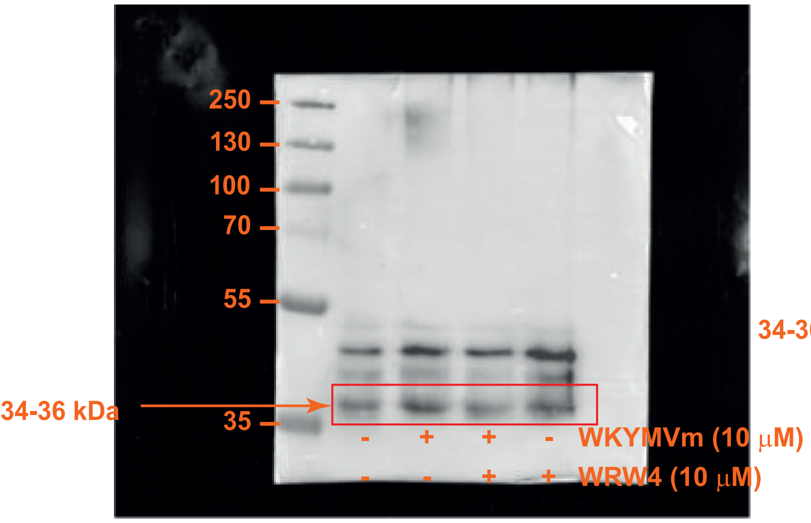

1E. WB: α-LAT1

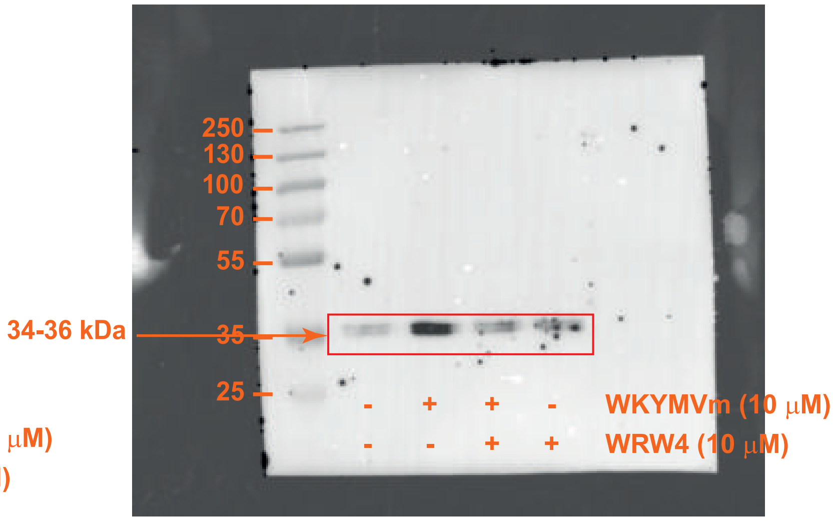

1D. WB: α-β-actin

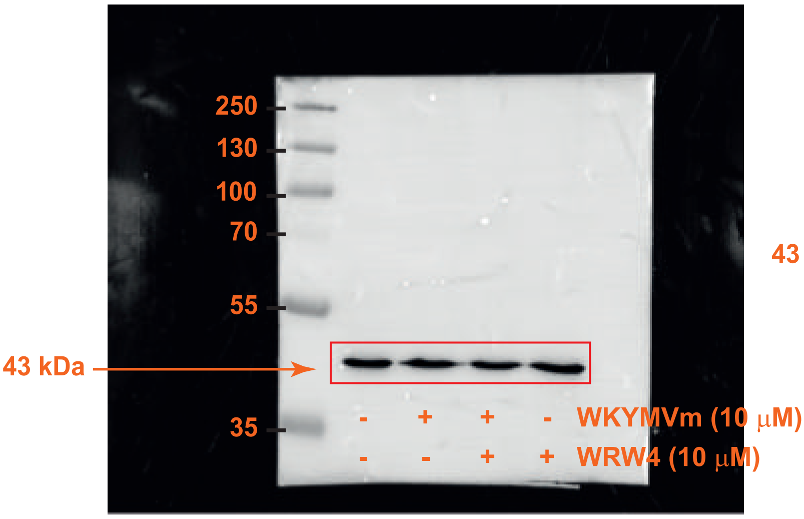

1E. WB: α-β-actin

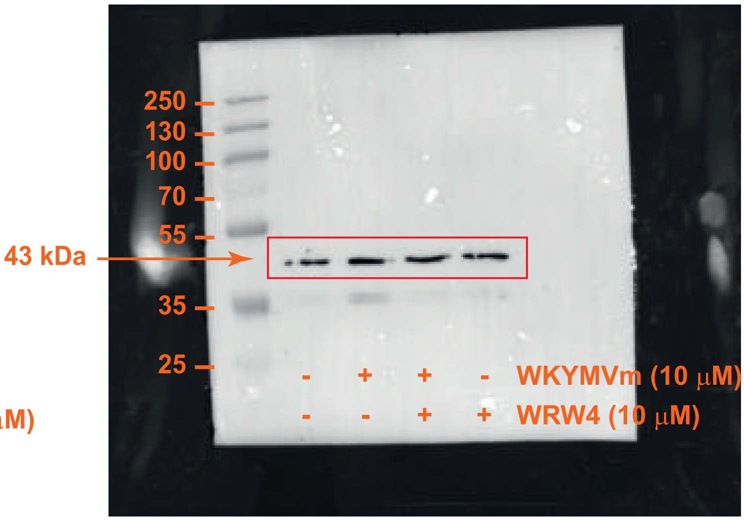

Supplementary Figure S2

2A.WB: α-LAT1

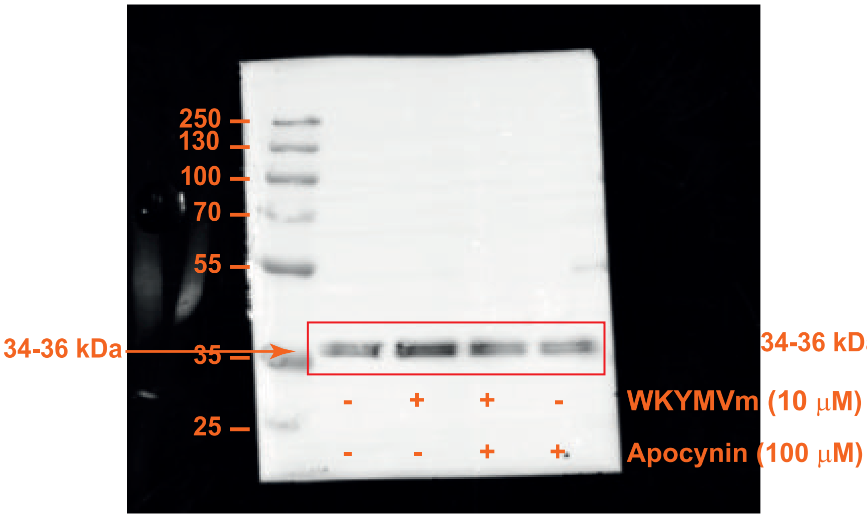

2B. WB: α-LAT1

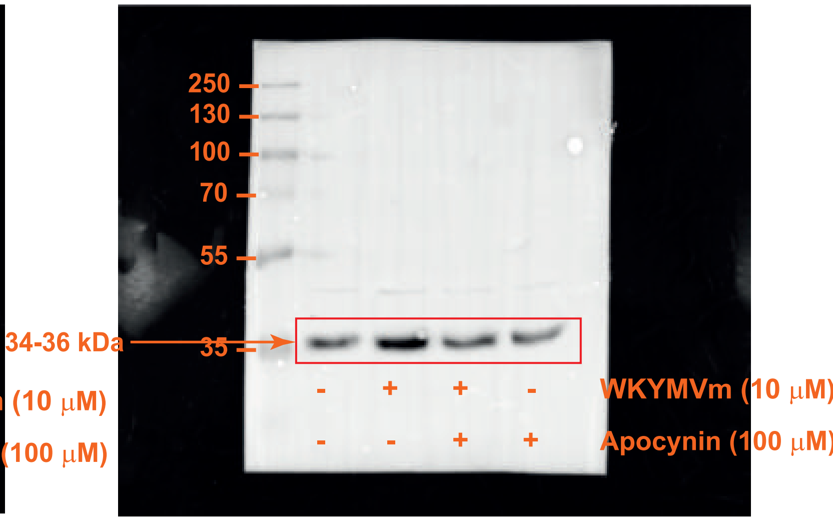

2A. WB: α-β-actin

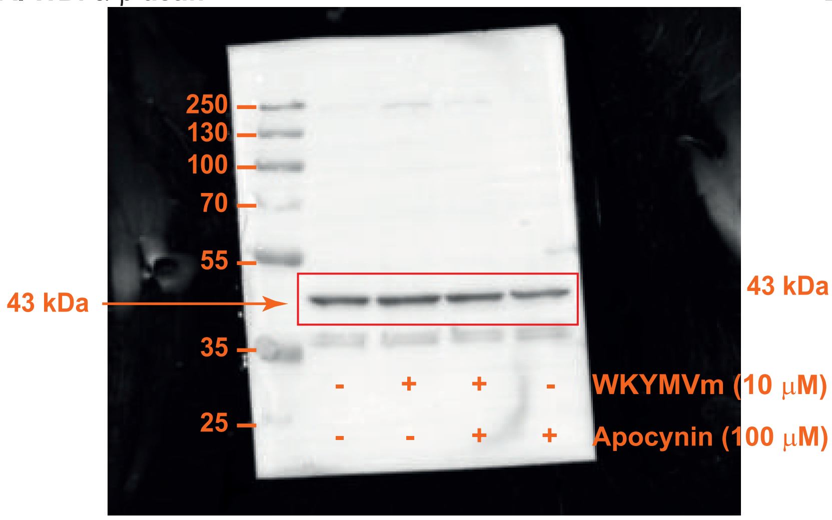

2B. WB: α-β-actin

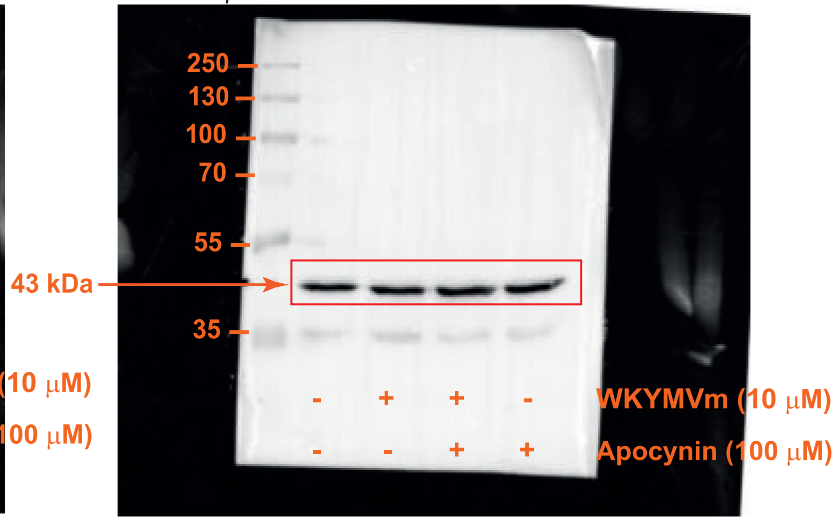

WB: α-LAT1

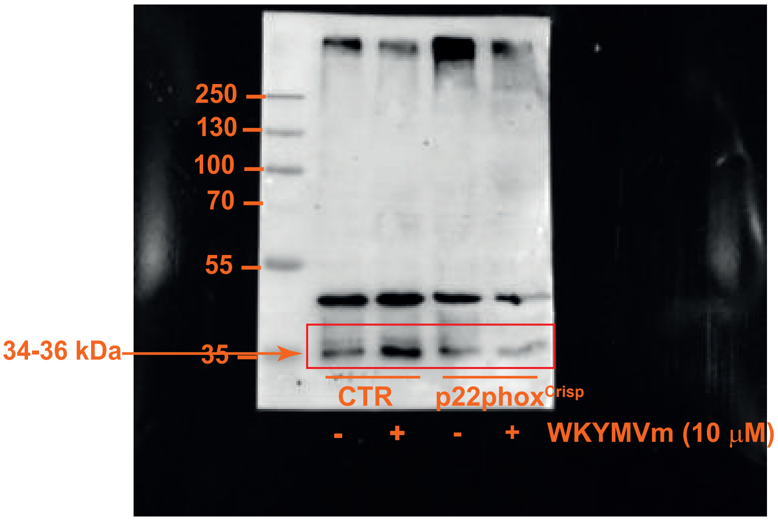

WB: α-β-actin

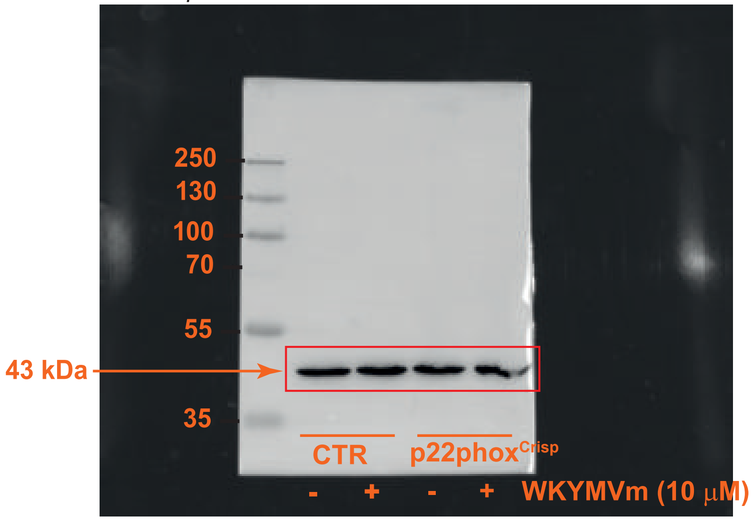

Supplementary Figure S3

3A. WB: α-CD98

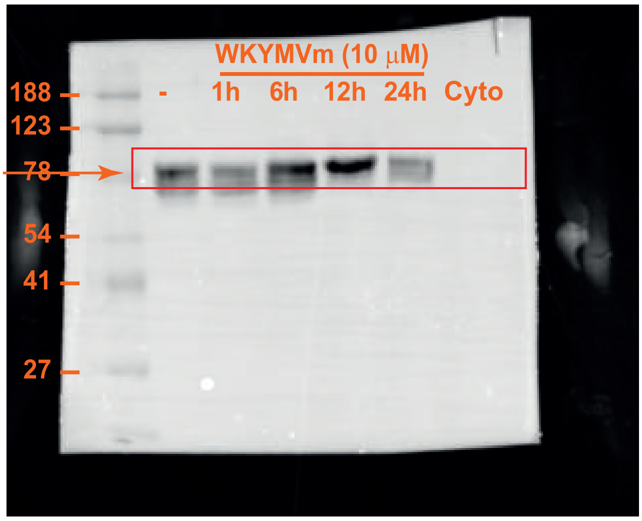

3B. WB: α-CD98

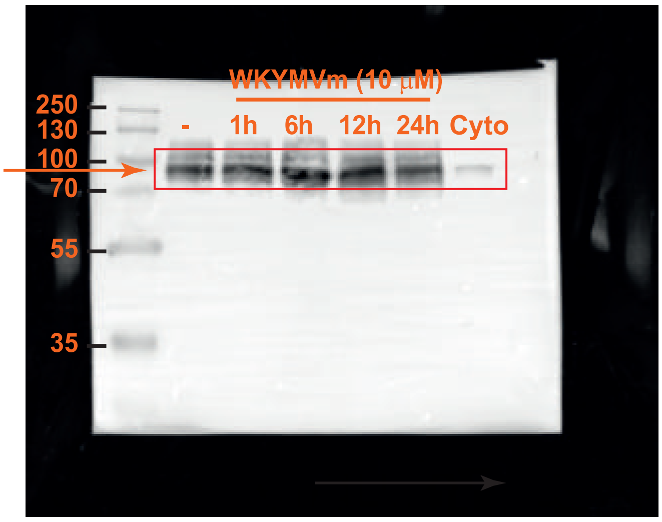

3A. WB: α-Na⁺/K⁺ ATPase

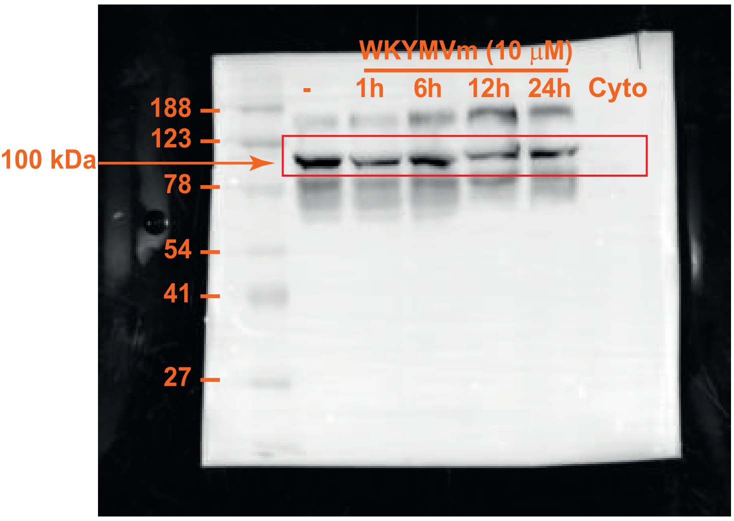

3B. WB: α-Na⁺/K⁺ ATPase

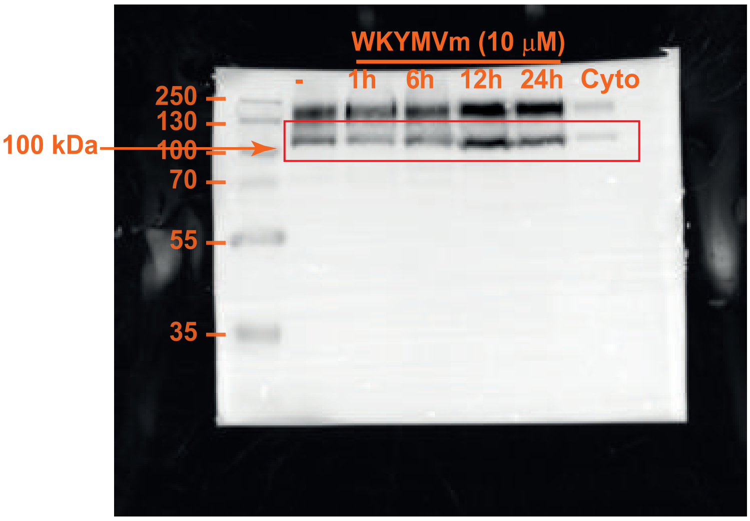

3A. WB: α-GAPDH

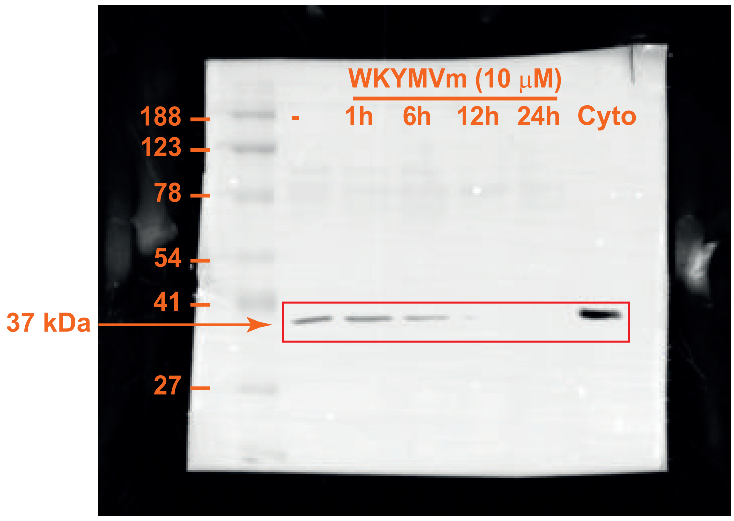

3B. WB: α-GAPDH

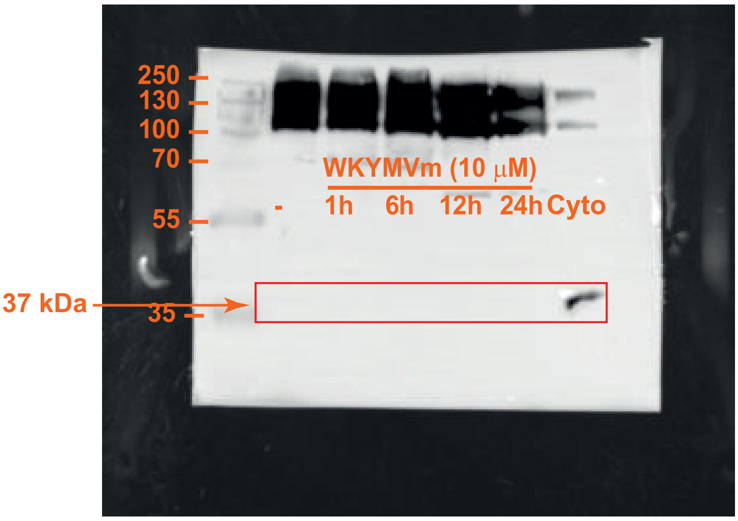

Supplementary Figure S4

5A. WB: α-p-S6K(Thr389)

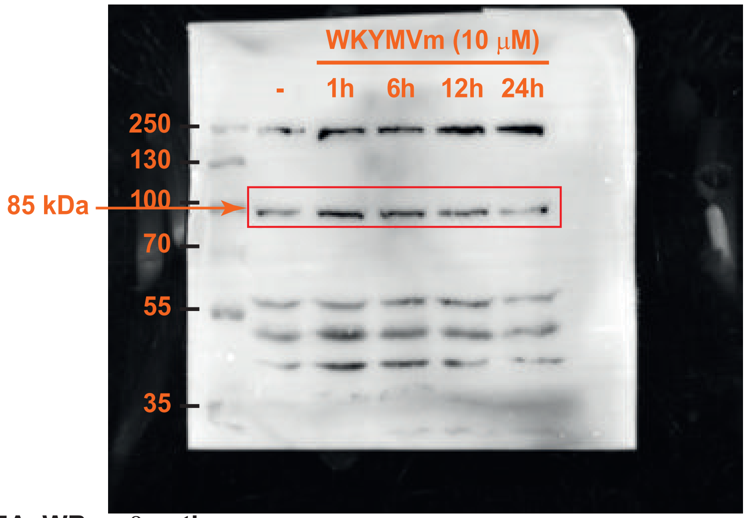

5B. WB: α-p-S6K(Thr389)

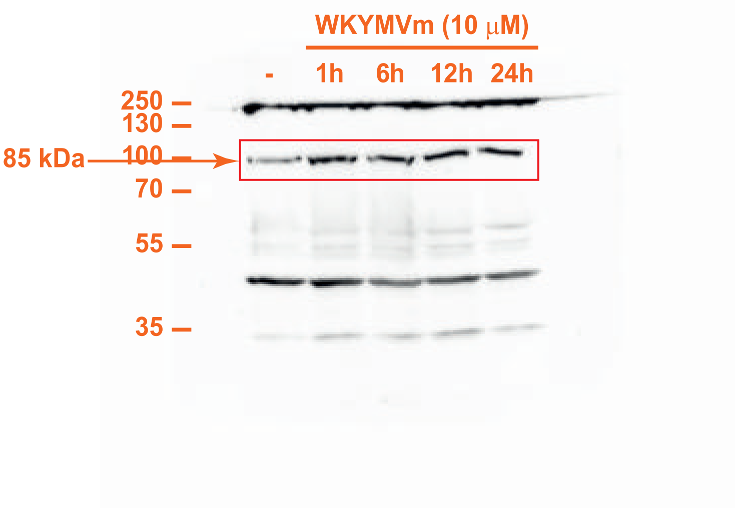

5A. WB: α-β-actin

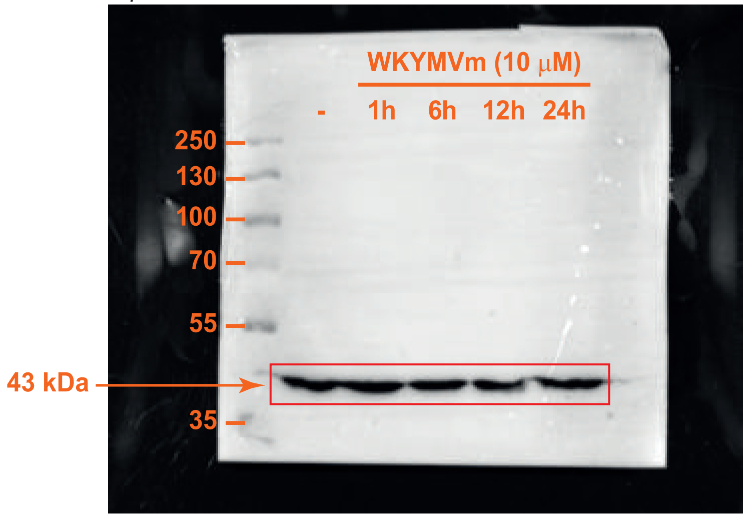

5B. WB: α-β-actin

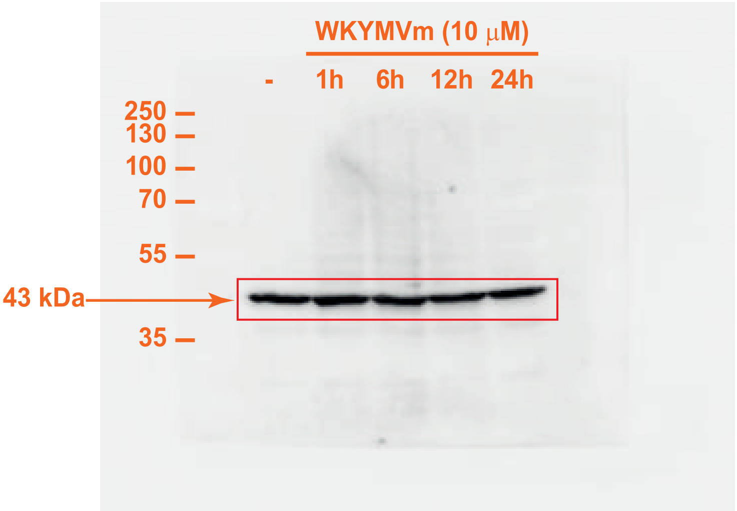

5C. WB: α-p-S6K(Thr389)

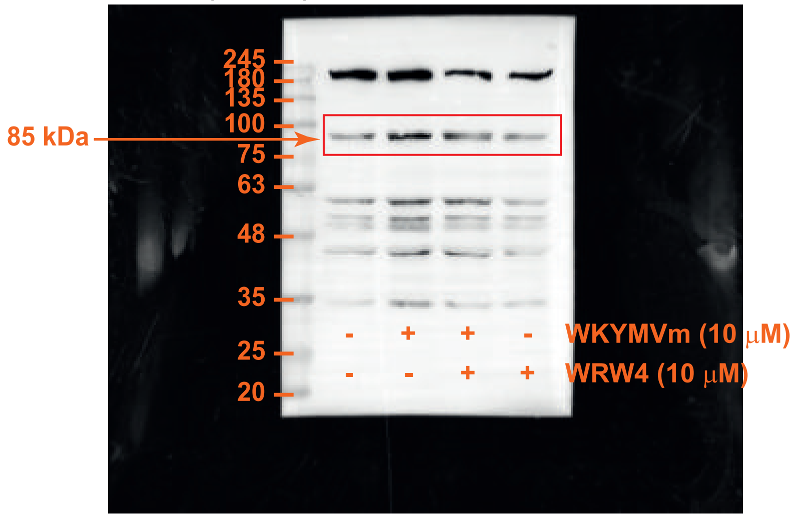

5D. WB: α-p-S6K(Thr389)

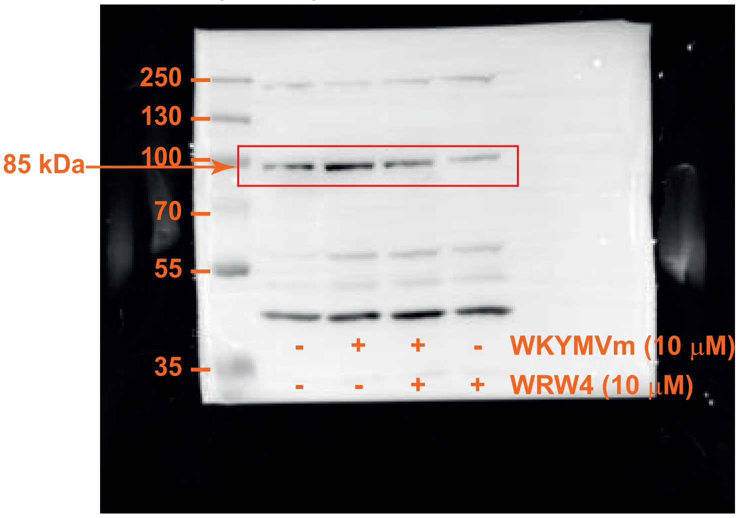

5C. WB: α-β-actin

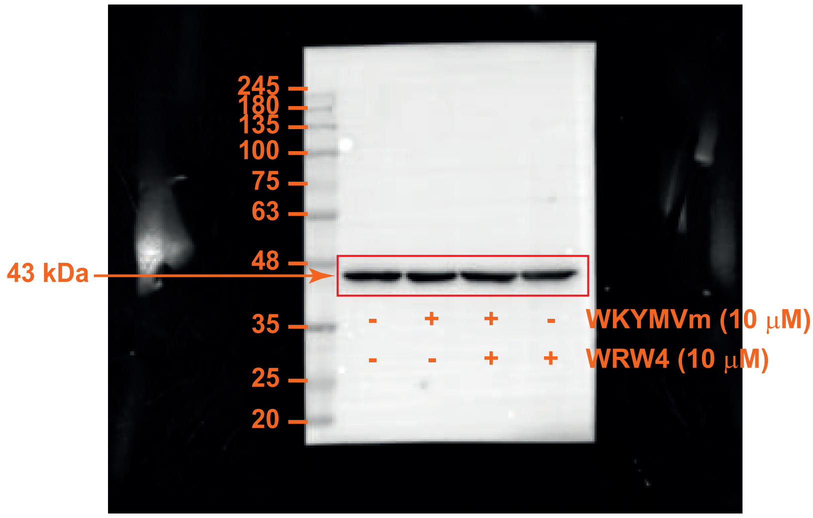

5D. WB: α-β-actin

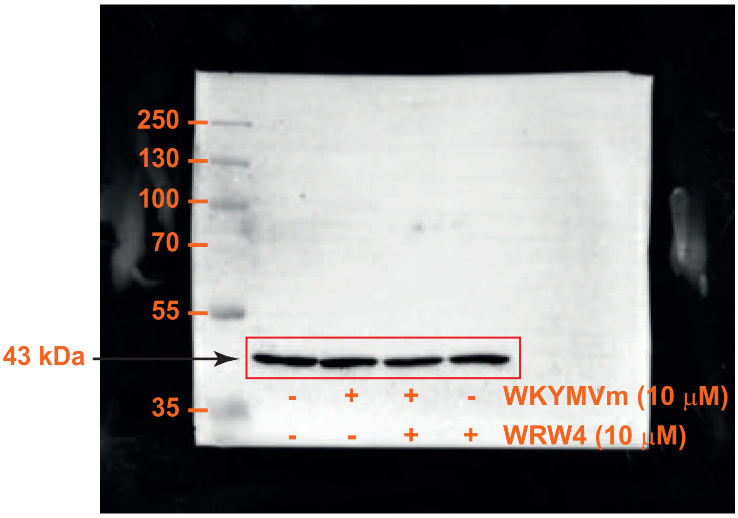

Supplementary Figure S5

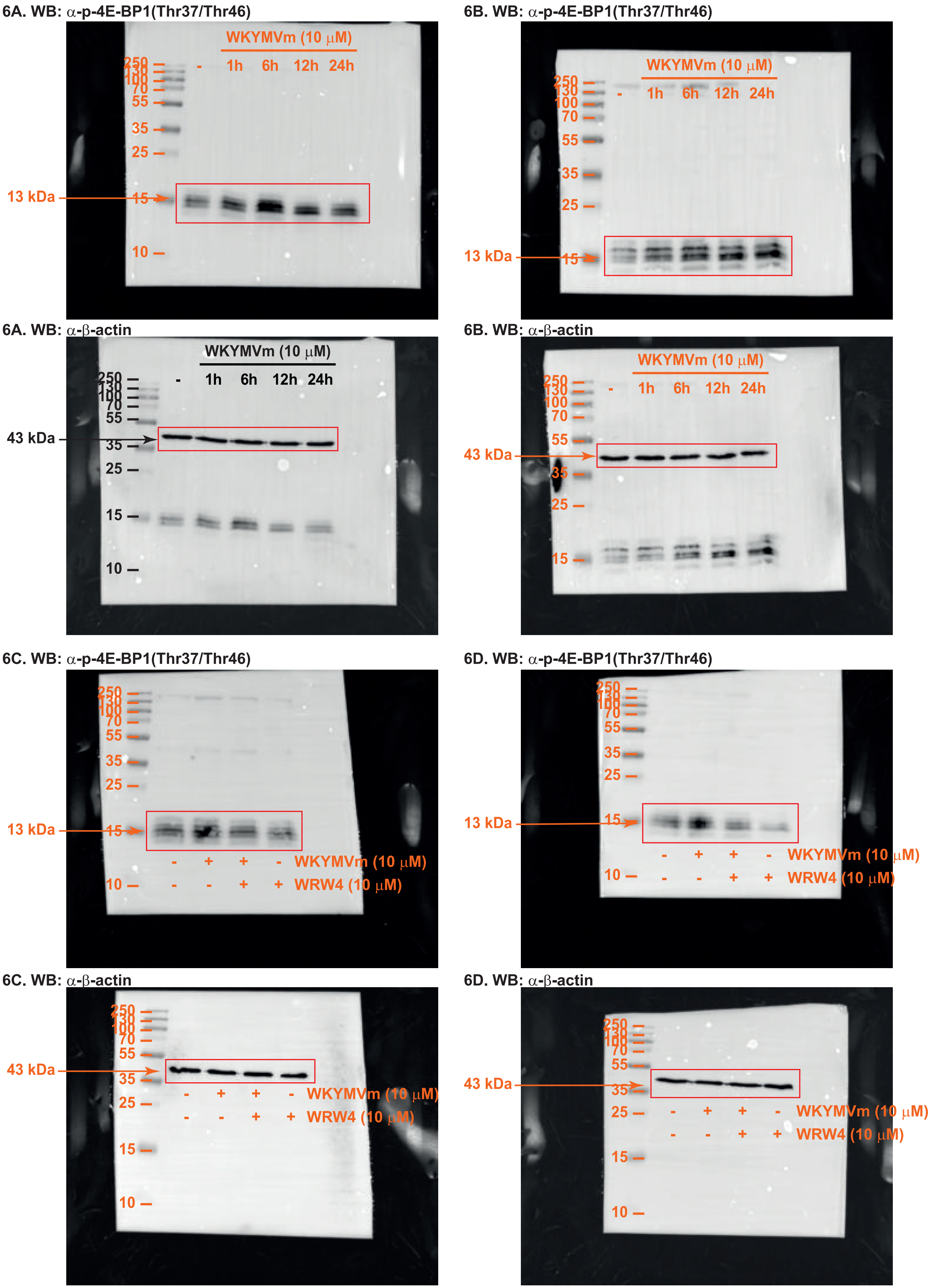

Supplementary Figure S6

7A. WB: α-p-S6K(Thr389)

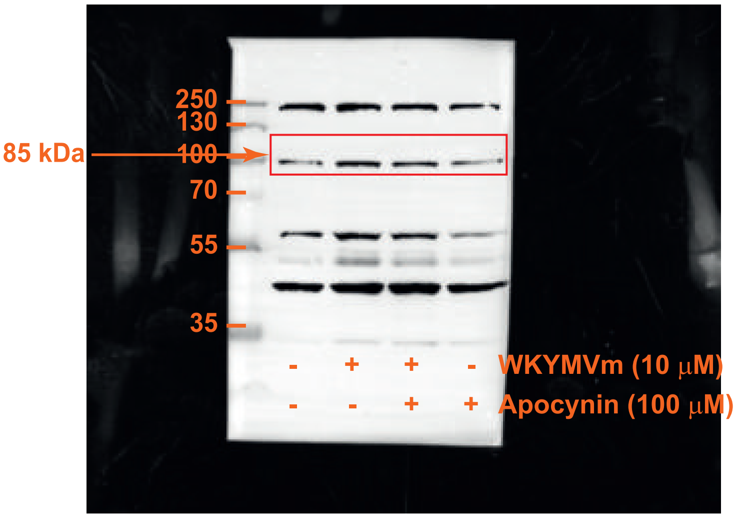

7B. WB: α-p-4E-BP1(Thr37/Thr46)

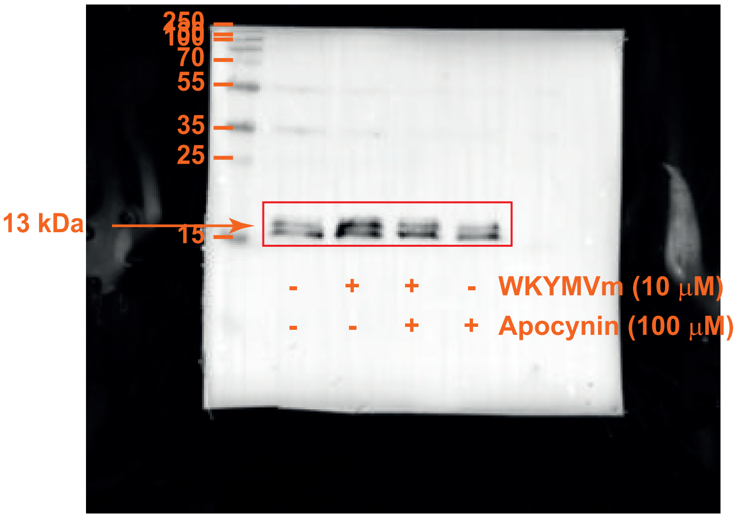

7A. WB: α-β-actin

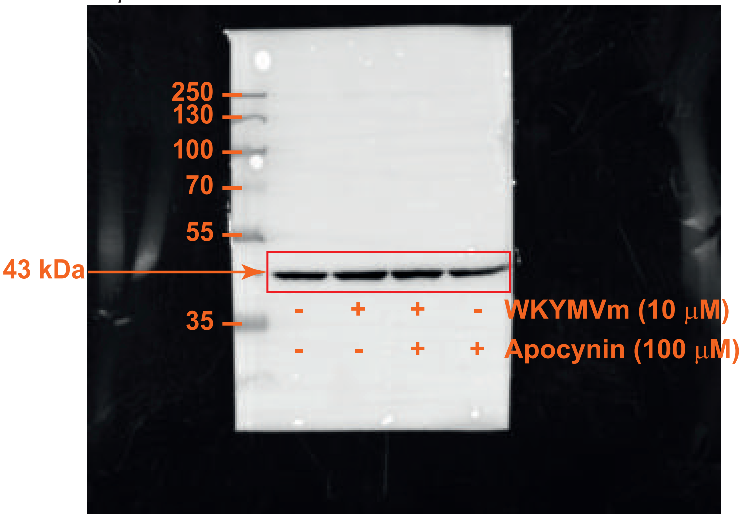

7B. WB: α-β-actin

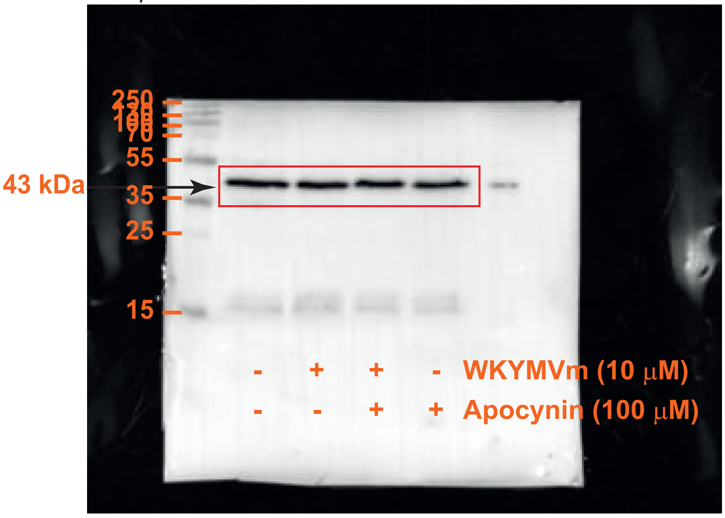

7C. WB: α-p-S6K(Thr389)

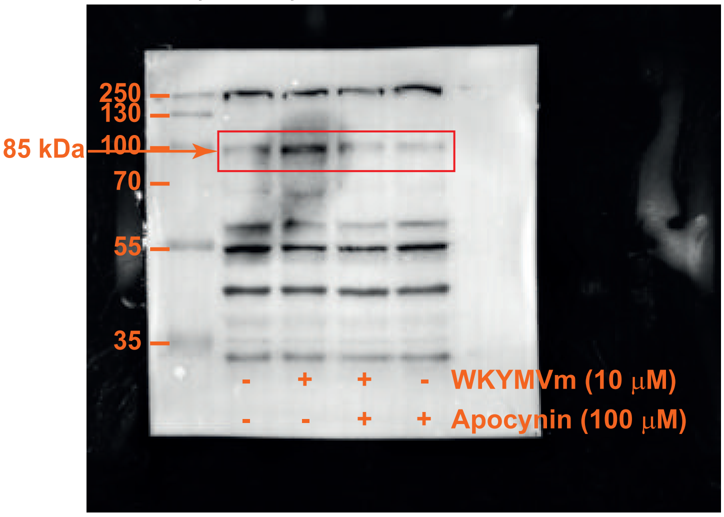

7D. WB: α-p-4E-BP1(Thr37/Thr46)

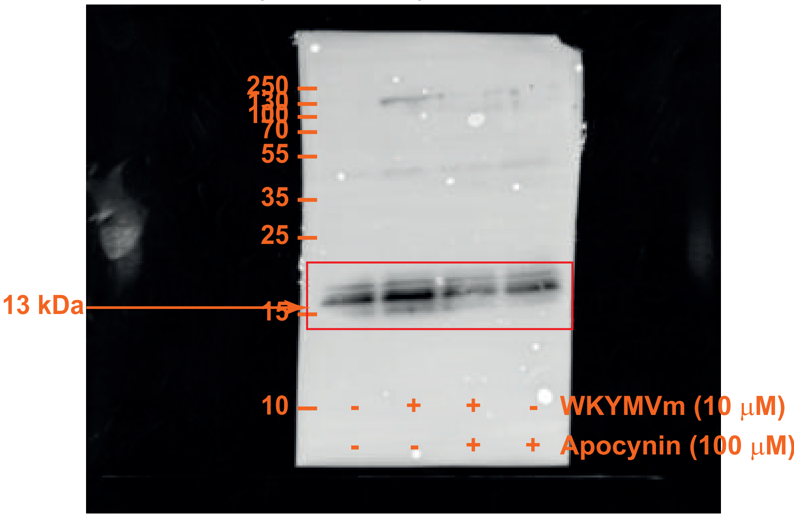

7C. WB: α-β-actin

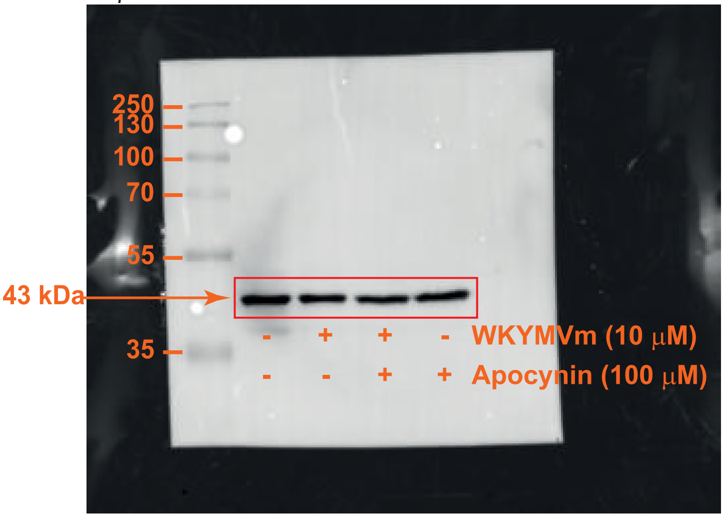

7D. WB: α-β-actin

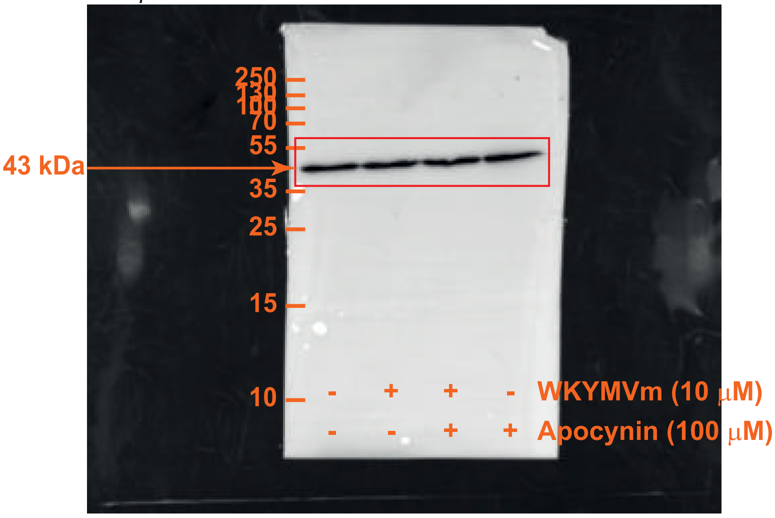

Supplementary Figure S7

7E. WB: α-p-S6K(Thr389)

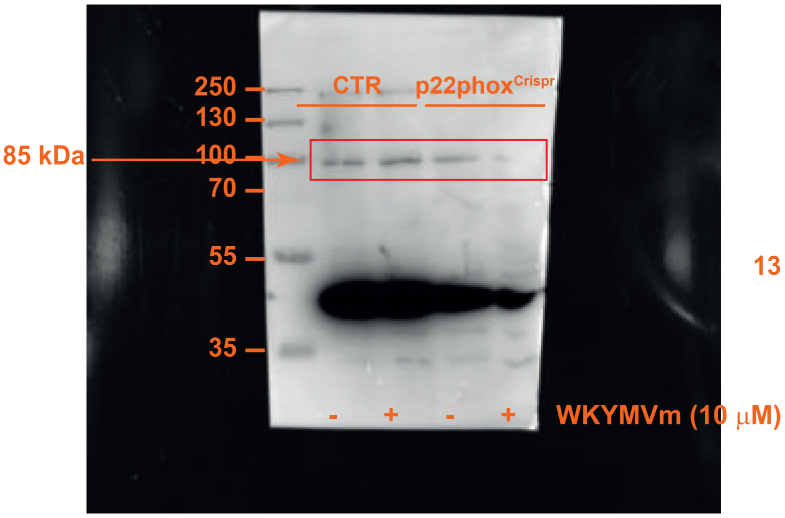

7F. WB: α-p-4E-BP1(Thr37/Thr46)

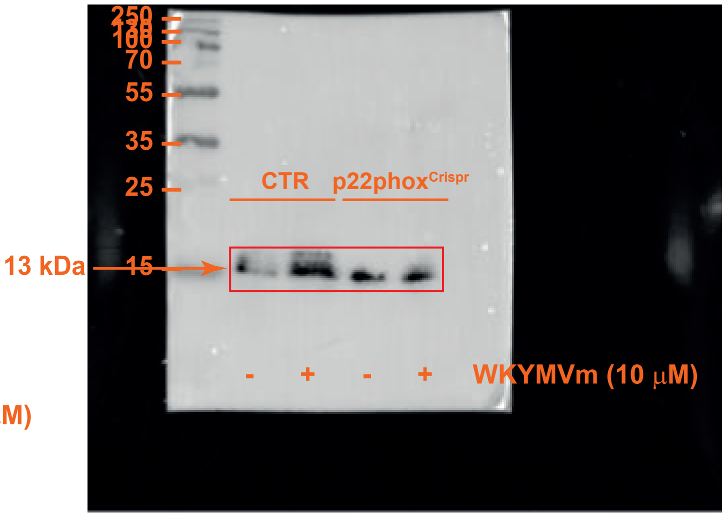

7E. WB: α-β-actin

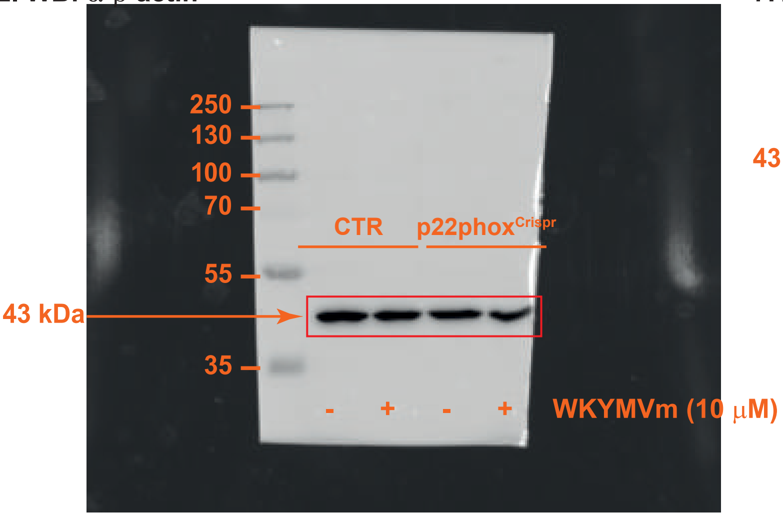

7F. WB: α-β-actin

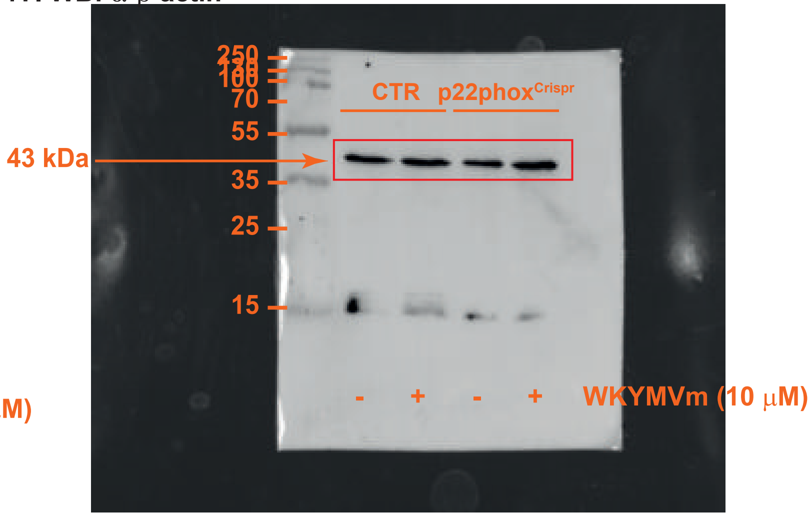

Supplementary Figure S8

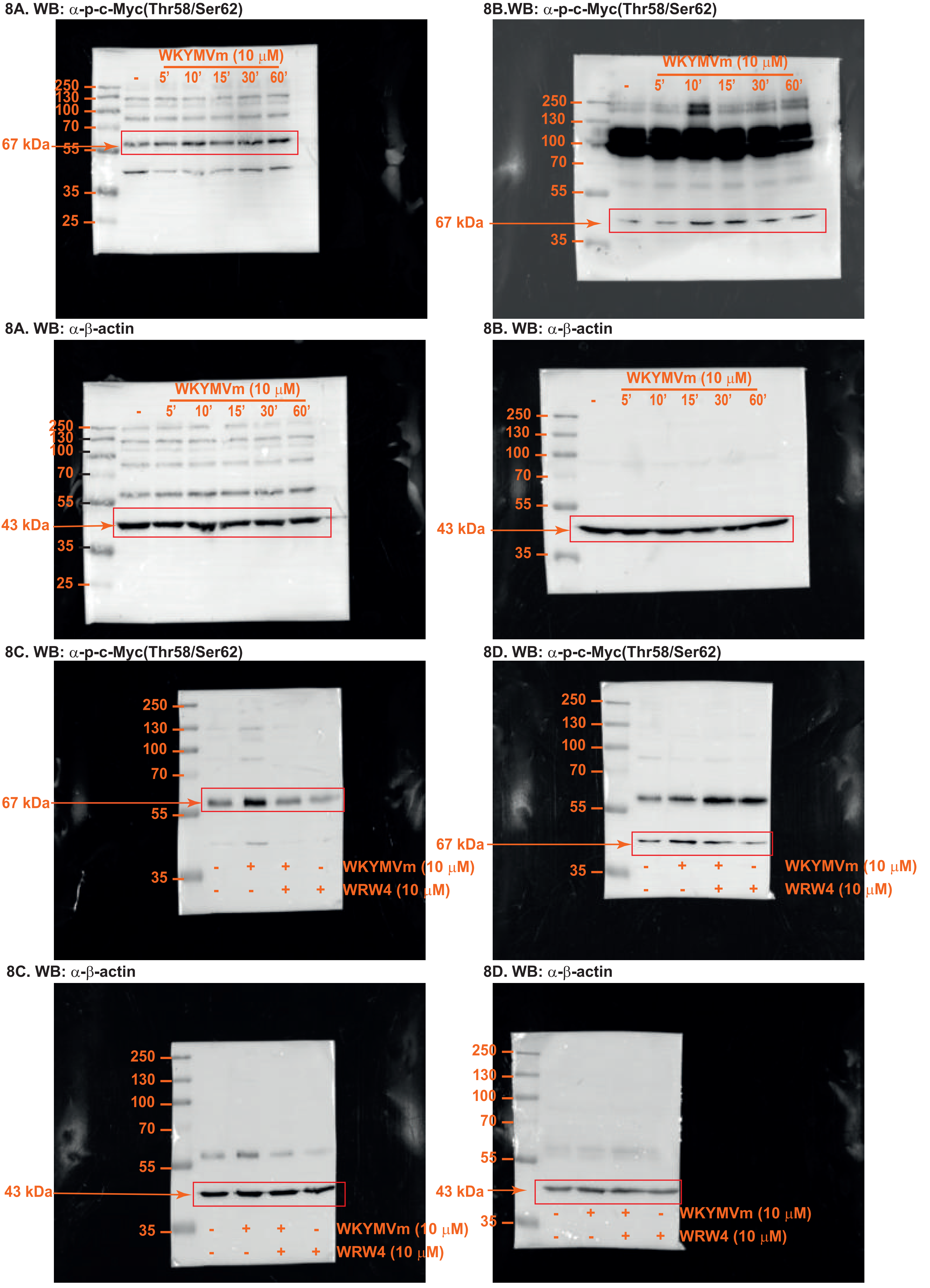

Supplement: Supplementary file 1 — Figure S1. Full blot Figure 1B‐E. WKYMVm is a FPR2 agonist; WRWWWW (WRW4) is a FPR2 antagonist. Figure S2. Full blot Figure 2A‐C. WKYMVm is a FPR2 agonist; Apocynin is selective inhibitor of p22phox. CaLu‐6‐control Crispr/Cas9 cells (CTR) and p22phox Crispr/Cas9 (p22phoxCrispr). Figure S3. Full blot Figure 3A and B. WKYMVm is a FPR2 agonist. Cytosolic fraction (Cyto). Figure S4. Full blot Figure 5A‐D. WKYMVm is a FPR2 agonist; WRWWWW (WRW4) is a FPR2 antagonist. Figure S5. Full blot Figure 6A‐D. WKYMVm is a FPR2 agonist; WRWWWW (WRW4) is a FPR2 antagonist. Figure S6. Full blot Figure 7A‐D. Full blot Figure 2A‐C. WKYMVm is a FPR2 agonist; Apocynin is selective inhibitor of p22phox. Figure S7. Full blot Figure 7E and F. Full blot Figure 2A‐C. WKYMVm is a FPR2 agonist; CaLu‐6‐control Crispr/Cas9 cells (CTR) and p22phox Crispr/Cas9 (p22phoxCrispr). [file FEBS-293-2637-s001.pdf]
